# Impact of two centuries of intensive agriculture on soil carbon, nitrogen and phosphorus cycling in the UK

**DOI:** 10.1016/j.scitotenv.2018.03.378

**Published:** 2018-09-01

**Authors:** Shibu E. Muhammed, Kevin Coleman, Lianhai Wu, Victoria A. Bell, Jessica A.C. Davies, John N. Quinton, Edward J. Carnell, Samuel J. Tomlinson, Anthony J. Dore, Ulrike Dragosits, Pamela S. Naden, Margaret J. Glendining, Edward Tipping, Andrew P. Whitmore

**Affiliations:** a Rothamsted Research, Harpenden, Hertfordshire AL5 2JQ, UK; b Rothamsted Research, North Wyke, EX20 2SB, UK; c Centre for Ecology & Hydrology, Wallingford, Oxfordshire OX10 8BB, UK; d Lancaster Environment Centre, Lancaster University, LA1 4YQ, UK; e Centre for Ecology & Hydrology, Bush Estate, Penicuik EH26 0QB, UK; f Centre for Ecology & Hydrology, Library Avenue, Lancaster LA1 4AP, UK

**Keywords:** Roth-CNP, Integrated model, Crops, Nutrient flux, Leaching

## Abstract

This paper describes an agricultural model (Roth-CNP) that estimates carbon (C), nitrogen (N) and phosphorus (P) pools, pool changes, their balance and the nutrient fluxes exported from arable and grassland systems in the UK during 1800–2010. The Roth-CNP model was developed as part of an Integrated Model (IM) to simulate C, N and P cycling for the whole of UK, by loosely coupling terrestrial, hydrological and hydro-chemical models. The model was calibrated and tested using long term experiment (LTE) data from Broadbalk (1843) and Park Grass (1856) at Rothamsted. We estimated C, N and P balance and their fluxes exported from arable and grassland systems on a 5 km × 5 km grid across the whole of UK by using the area of arable of crops and livestock numbers in each grid and their management. The model estimated crop and grass yields, soil organic carbon (SOC) stocks and nutrient fluxes in the form of NH_4_-N, NO_3_-N and PO_4_-P. The simulated crop yields were compared to that reported by national agricultural statistics for the historical to the current period. Overall, arable land in the UK have lost SOC by −0.18, −0.25 and −0.08 Mg C ha^−1^ y^−1^ whereas land under improved grassland SOC stock has increased by 0.20, 0.47 and 0.24 Mg C ha^−1^ y^−1^ during 1800–1950, 1950–1970 and 1970–2010 simulated in this study. Simulated N loss (by leaching, runoff, soil erosion and denitrification) increased both under arable (−15, −18 and −53 kg N ha^−1^ y^−1^) and grass (−18, −22 and −36 kg N ha^−1^ y^−1^) during different time periods. Simulated P surplus increased from 2.6, 10.8 and 18.1 kg P ha^−1^ y^−1^ under arable and 2.8, 11.3 and 3.6 kg P ha^−1^ y^−1^ under grass lands 1800–1950, 1950–1970 and 1970–2010.

## Highlights

• Roth-CNP model estimates C, N and P cycling within the UK agriculture for 1800–2010.
• Simulated crop yields were comparable to the yields of UK's agricultural statistics.
• Simulated SOC stock decreased under arable and increased under improved grassland.
• Simulated N and P losses increased under both arable and grasslands.
• Results shows the effect of local agriculture in a larger context of space and time.

## Graphical abstract

**Figure:**
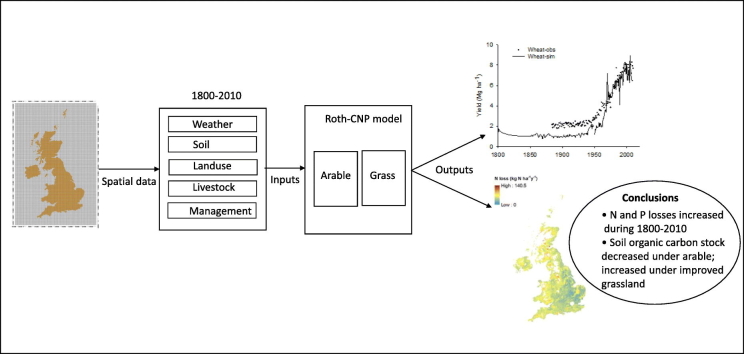

## 1 Introduction

Agriculture in the United Kingdom (UK) has a long history of human settlement and development which dates back to 6000 years ago when humans began domesticating plants and animals in Neolithic times (Edwards and Hirons, 1984; Woodbridge et al., 2014). By 900–700 BCE, settled agriculture was established in the UK with crop rotations, pasture and coppiced woodlands. By 100–350 CE, natural forest was largely cleared with large estate-based farming systems with cattle, sheep and arable production. The UK's countryside then further changed dramatically with the majority of the population living in small farmsteads under subsistence farming. By 1300 CE, increasing demand for food brought the subsistence farming system under huge pressure because of increasing population as the land area available for agriculture was already in use. However, between 1300 and 1800 average crop yields increased in the UK due to improvements in crop management such as mixed husbandry (by combining crop and livestock), grass and arable rotation, crop rotation by including fallow and legumes leading to a British agricultural revolution during 1700–1850 (Allen, 1999; Allen, 2008; Overton, 1996). With the industrial revolution in 1850s, technological improvements also happened in the agricultural sector, for example, switching from draught animals to machines in early 1900s (Whetham, 1970). Much of the agricultural growth during this period came about as a result of increases in the area of crops and grass, which peaked in mid 1880s. After this, the agricultural area underwent a steady decline as farms became more intensive and the availability of labour diminished. During the second half of the 20th century (Musel, 2009), agricultural intensification driven by new high yielding varieties, mineral fertilizer application, chemical pest control and improved methods of cultivation (Marks and Britton, 1989) led to increase in agricultural production many-fold. Per-hectare yields of wheat almost tripled whilst barley, potato yields and milk yields per cow more than doubled (DEFRA, 2014; Marks and Britton, 1989). The total cattle population increased sharply after the middle of 20th century although there has been a decline since 1974. About 170 million ton of animal excreta (slurry) are produced annually in the UK. In terms of farm inputs, mineral nitrogen (N) fertilizer used in the UK increased five times between 1950 and 1978 (Cooke, 1980). Greater use of N and P fertilizers during this period has led to an increased loss of these nutrients into our rivers and ground water through leaching, runoff (Hood, 1982; Hooda et al., 2000), and increased atmospheric emissions of ammonia, nitrous oxide and other reactive N compounds. Agricultural land contributes 70% and 28% of the N and P load to the UK waters (Hunt et al., 2004; White and Hammond, 2007). Losses of these nutrients are associated with excessive or poorly timed applications of N or P or both (Dungait et al., 2012). Pretty et al. (2000) calculated the annual external cost of agriculture for the UK in 1996 as £2343 M (£208/ha), with the major costs associated with contamination of drinking water by pesticides, nitrate and phosphate and increased greenhouse gas (GHG) emissions, soil erosion and organic carbon losses.

Numerous spatially-variable, interacting factors such as land-use, vegetation type, weather, catchment topography and total nutrient inputs over time determine the nutrient stocks and fluxes at a farm, landscape or catchment scale. For example, nutrient concentrations in groundwater under agricultural land have been found to be several times higher than that under semi-natural vegetation (Nolan and Stoner, 2000). Growing vegetables and crops such as potatoes and oilseed rape intensively has led to high rates of nitrate leaching (Stuart et al., 2011). Nutrient concentrations in ground water have been found to be highly variable and related to changes in the weather (Rozemeijer et al., 2009) and increased as a result of land-use change (Whitmore et al., 1992). There is a strong influence of catchment slope on water quality due to slope-dependent seasonal waterlogging, which determines the fate of dissolved substances produced within and moving through the catchment (D'Arcy and Carignan, 1997). Temporal dynamics of these nutrients depend on the relative occurrence of the nutrients in different pools at different points in time. Nutrients are retained during the dry summer months as a result of bioaccumulation and adsorption in case of P, and during the wetter autumn to spring periods, these nutrients are released and transported from the floodplain into the river channel (Bowes et al., 2005).

Understanding the processes that have led to the build-up of C, N, and P in soil, ground water and surface water from the past to the present is essential to understand how to manage the supply and utilization of these nutrients into the future. This will contribute to the long-term goal of achieving a sustainable agricultural system by increasing or maintaining crop yields whilst minimising impacts on other ecosystem services (Powlson et al., 2011). It is also important to understand how these nutrient cycles (between atmosphere, terrestrial ecosystems including agriculture and hydrological systems) operate at large spatial scales across the whole UK in response to climate change and management options. A model that can summarise essential processes of soil and plant growth and their interactions and that can be applied over long timescales with readily-available driving data (climate, land-use, nutrient inputs) is essential to investigate the temporal and spatial responses in soil macronutrients at the national scale. Such a study should help both farmers and policy makers to see the effect of agriculture at the local scale in a larger context of space and time.

There are many agroecosystem models in the literature that can simulate the C, N, and P cycling under crop and grassland systems at the field scale EPIC (Jones et al., 1991); DNDC (Li et al., 1992), APSIM (McCown et al., 1996); DAYCENT (Parton et al., 1998); CropSyst (Stöckle et al., 2003); DSSAT (Jones et al., 2003). A recent review assessed the comprehensiveness of underlying processes in nine widely used C and N models found that one of the major weakness of these models is their scalability over time and space (Brilli et al., 2017). To describe soil C and N dynamics at higher spatial and temporal scales we need models of lower complexity and with longer timesteps (McGill, 1996; Manzoni and Porporato, 2009). In the literature, we can find many process-based models that were applied at national or global scale to estimate C stocks for terrestrial systems (Al-Adamat et al., 2007 (Century and RothC); Kamoni et al., 2007 (Century and RothC); Ogle et al., 2003(IPCC method); Smith et al., 2010 (ECOSSE); Tian et al., 2015 (MsTMIP); Wang et al., 2017 (RothC). Many studies estimate N and P balances at the national or global scale using spatially explicit data on crop area, livestock population, fertilizer input rates and empirical models to estimate nutrient stocks and fluxes (Smil, 1999; Lesschen et al., 2007; Liu et al., 2010; Pathak et al., 2010; MacDonald et al., 2011; Bouwman et al., 2013; Cui et al., 2013). However, only a few studies use process models to simulate coupled C, N and P models to estimate the stocks and fluxes of these macronutrients for the terrestrial systems at the national or global scale (Wang et al., 2010; Zaehle, 2013). However, in all these models, the concept of the cropping system is often simplified by omitting details of crop management, such as rotation (Leenhardt et al., 2010). In this study we use a simplified agricultural model that aggregates processes at a monthly timestep as compromise between detailed process modelling and computation time when run at 5 × 5 km grid for the UK. We chose RothC (Coleman et al., 1997) model extended for N and P (Coleman et al., 2017) as the model to describe the C, N and P dynamics because of its simplicity and its applicability in the agricultural system in the UK. Plant (both crop and grass) growth processes were based on the LINTUL model (Shibu et al., 2010; Wolf, 2012) and were simplified to suit the monthly timestep of RothC. Atmospheric and hydrological models are run at finer timesteps and atmospheric N deposition, evapotranspiration, runoff, drainage and erosion scaled up to the monthly timestep.

**Fig. 1:**
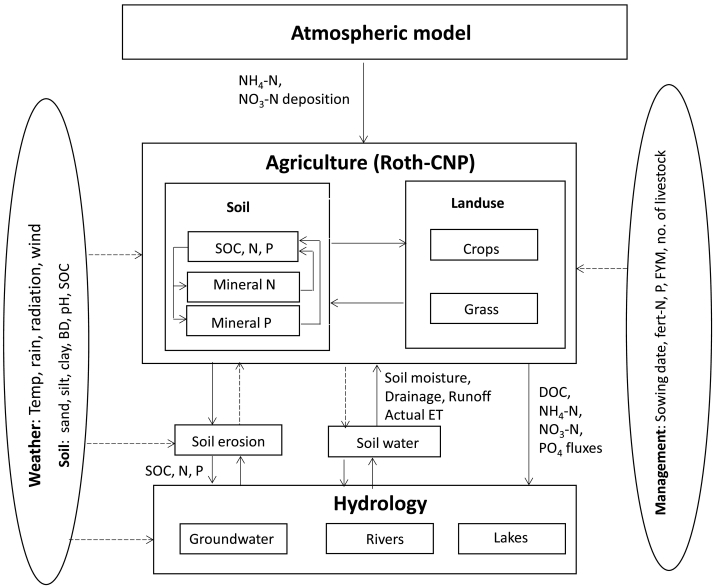
Schematic diagram showing the structure of Roth-CNP model interacting with components (atmospheric, hydrology, soil water and soil erosion models) of the Long-term Large Scale Integrated model (LTLS-IM) (Arrows indicate material and information flow; dotted arrow indicate information flow only). (Abbreviations: BD: bulk density; SOC, N, P: Soil organic carbon, nitrogen and phosphorus; ET: evapotranspiration; DOC: dissolved organic carbon).

This paper estimates C, N and P pools, pool changes, their balance and the nutrient fluxes exported from arable and grassland systems in the UK during the historical to current period (1800–2010) using an agricultural model that was developed as part of an integrated model to analyse and simulate long-term and large-scale (LTLS) interactions of C, N and P in the UK land, freshwater and atmosphere (http://www.ltls.org.uk/). This integrated model is referred here as LTLS-IM (Bell et al., in prep), which loosely couples terrestrial (semi-natural and agricultural), hydrological and hydro-chemical models and driven by atmospheric deposition (Fig. 1). Our emphasis in this paper is to present this integrated modelling approach with a major focus on estimates of UK-wide historical yield, SOC changes and nutrient fluxes.

## 2 Methodology

This study focuses on the CNP stock changes in soil in the agricultural system driven by the environmental variables, plant and livestock management. At a spatial scale of a 5 × 5 km grid, we did not consider field or farm scale dynamics or exchange of biomass or nutrients between the grid cells. We assume that lateral flow of nutrients across farms within a cell or between cells will be a ‘continuous’ process and will be balanced by the influx and the outflux within each cell. The agricultural model referred to as Roth-CNP model was developed by simplifying the Landscape Model (LM) (Coleman et al., 2017) to an appropriate level of detail. The LM which works on a daily timestep, simulates the biophysical processes of an agroecosystem at the field/farm scale taking into account the spatial interactions between the fields or farms across a landscape. The Roth-CNP model presented here aggregates the essential processes within the LM on a monthly timestep without any spatial interactions between the spatial units. We briefly describe here the main features of Roth-CNP model together with any major changes from the LM (Appendix I). In Roth-CNP, we use the same parameters as the LM but adapting for a monthly timestep. We tested the Roth-CNP model using the data from Broadbalk and Park Grass long-term experiments (LTEs) at Rothamsted (*http://www.era.rothamsted.ac.uk**/*), South-East England before undertaking the historical simulation for a continuous period from 1800 to 2010 for the whole of the UK. For these simulations, the whole land area in the country was divided into 5 km × 5 km cells on a square grid with improved grass present in in 91% and arable land in 76% of the grids cells (see SI, Fig. S2.1).

### 2.1 Model description

The Roth-CNP model (Fig. 1) has two major subunits: soil and landuse. In the soil module, the soil profile (can be of any depth, but in this study, it ranges from 30 cm to 150 cm) is divided into three layers. Depths of soil layers can be variable, but for this application the first and second layers were set to 15 cm each to enable a spatial comparison of CNP pools as most of the soil management activities affect the top 30 cm. The depth of the third layer is variable depending on the actual soil profile, which varies spatially across the UK. The soil unit consists of organic C, N and P, mineral N and P modules. Variables such as actual evapotranspiration (AET), soil drainage, runoff and soil moisture are treated as inputs that are calculated by a hydrological model, which is a simplified version of the G2G model (Bell et al., 2009). However, potential evapotranspiration (PET) for each landuse was estimated in a crop module (as it varies with crop type and developmental stage of the crop) based on the Penman's method (Penman, 1948). The PET estimated by the crop model was compared to the PET estimated by the hydrology model (using MORECS PET for grass assuming variable leaf area index (LAI) for summer and winter (Hough and Jones, 1997) for a few selected sites and were found to be comparable (not reported here) with a difference in PET of up 5% in winter to 12% in summer. The hydrology model within the LTLS-IM calculates components of the water balance (runoff, drainage, AET and soil moisture) for each 5 × 5 km grid-cell in the UK. Soil moisture for the entire profile (mm of water/profile depth) was used to estimate moisture content in each soil layer within the Roth-CNP model. Soil organic carbon dynamics inherited from the RothC model has been described elsewhere (Coleman and Jenkinson, 1999; Smith, 2000; Jenkinson and Coleman, 2008). The model was extended for organic N and P with similar pool structures as that for carbon determined by the C/N or C/P ratios of the incoming organic materials for Decomposable Plant Material (DPM), Resistant Plant Material (RPM), and a fixed C/N or C/P ratios for Microbial Biomass (BIO) and Humified Organic Matter (HUM) (Coleman et al., 2017). Additional temporary pools of dissolved organic carbon (DOC), nitrogen (DON) and phosphorus (DOP) were created in the model in order to estimate the loss of dissolved organic C, N and P that enters soil solution. These pools are not linked to the main C, N, P pools and become active only when manures are added to the soil. In agricultural soils, added organic amendments such as farm yard manure (FYM), slurry and other animal manures are the major sources that contribute to DOC (Bhogal et al., 2010). Since we could find little information on the export of DOC from soils under agriculture, we assume that soil organic carbon (SOC) itself contributes only a negligibly small amount to DOC and therefore, its loss from agricultural lands was ignored in this study. In the model, we assume that when organic substrates are added, a fraction (FYM-4.6%; slurry-51%, and poultry manure- 6.6%) of these goes directly to the DOC, DON and DOP pools (Bhogal et al., 2010) and is lost by leaching and/or runoff immediately before the reminder enters the SOC, SON and SOP pools.

Mineral N and P species exist in single (vertically integrated) stores without partitioning them between different soil layers to co-exist with the dynamics of soil water which estimates the water balance for the whole profile. All the N and P mineralised from soil organic matter (SOM^1^) in the three soil layers is transferred to these mineral nutrient stores. Mineral N consists of NH_4_^−^N and NO_3_^−^N pools and mineral P includes *available* and *fixed* pools. Mineral N dynamics comprises N inputs (through atmospheric deposition, biological N fixation, fertilization), transformations (nitrification and denitrification) and losses (through plant uptake, denitrification, runoff, leaching and erosion) (Coleman et al., 2017). Similarly, P dynamics comprises P inputs from fertilizers, chemical P fixation and release, crop uptake, runoff, leaching and erosion. P contribution from weathering is not simulated in the model separately, but it is considered as a part of the *fixed* pool. Dynamic processes leading to an equilibrium between P in fixed and available pools are described elsewhere (Coleman et al., 2017).

The rate of nitrification and denitrification depends on the relative nitrification (0.99 month^−1^) and denitrification rates (0.20 month^−1^), soil temperature, moisture and pH (Coleman et al., 2017). In the model we assume that biological N fixation (BNF) occurs only in grassland systems and on an average about 30% of grassland is a leguminous clover mix and can fix N biologically (Lüscher et al., 2014; Sanderson et al., 2013). In the model, BNF rate is calculated as a function of potential maximum N fixation rate and the rate modifying factors for temperature (*f*_*T*_),soil moisture (*f*_*m*_) and inorganic N (*f*_*N*_) (Liu et al., 2013).

(1) $${\mathrm{Nfix}}_{\mathrm{rate}}={\mathrm{Nfix}}_{\max}\;\;f_{T}\;\;f_{m}\;\;f_{N}$$

where *Nfix*_*rate*_ and *Nfix*_*max*_ are the actual and maximum rates of BNF (g N m^−2^ month^−1^).

Potential maximum BNF rate depends on the live shoot biomass (g DM m^−2^), fixation rate per unit standing biomass (g N g^−1^ DM month^−1^) and root growth rate (g DM month^−1^). See Liu et al. (2013).

Increases in mineral N (NH_4_N and NO_3_N) concentration reduce the BNF rate in the model and we assume N that is fixed is directly transferred to the NH_4_N pool.

Mineral N and P losses occur either through runoff (in water phase) or through soil erosion (particulate) and leaching. Loss of these nutrients through runoff depends on both the nutrient (NO_3_N, available P) concentration (kg mm^−1^) at the surface (calculated as a function of depth) and the runoff (mm of water month^−1^). Since nutrient distribution in the profile may depend on the soil water and other soil profile characteristics (e.g. soil organic matter distribution, P weathering) it will be difficult predict their distribution in the profile over time. We assume nitrogen largely follows the pattern of SOC distribution with more NO_3_-N on the surface compared to the subsurface. Therefore, to estimate the NO_3_-N content for the 15 cm layer, we used an exponentially decreasing function, which will decrease with increase in soil depth. Leaching depends on the nutrient concentration (kg mm^−1^) in the soil solution and the drainage rate (mm of water month^−1^). Estimated rates of runoff and drainage were input from the hydrology model (see Section 2.2.5).

**Fig. 2:**
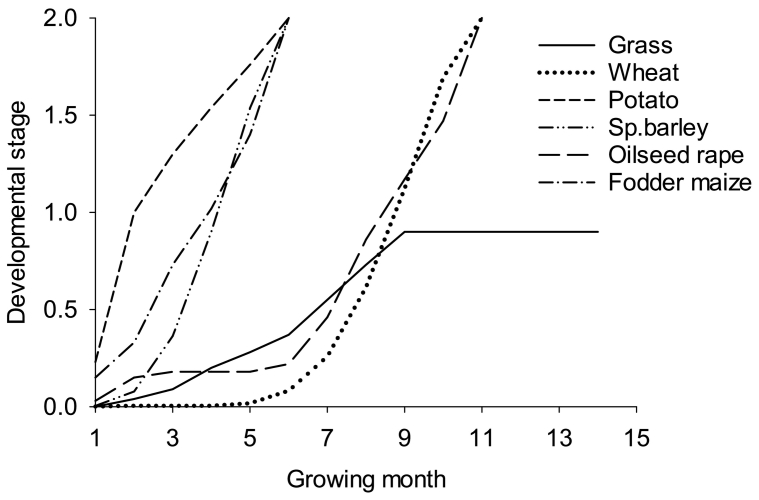
Developmental stage (DVS), which is a function of temperature sum or growing degree days and includes the effect of vernalisation and/or photosensitivity of the crop, estimated for different crops and grass as a function of their growing months (for winter wheat, 1–11 growing months = October–August; for potato, 1–5 growing months = April–August; for spring barley, 1–6 growing months = March–August; for Oilseed rape, 1–11 growing months = September–July; for fodder maize, 1–4 growing months = May–August, for grass, growing months are indefinite. Growing months are based on MAFF (1998)).

A generic plant growth model, which uses the light use efficiency (LUE, g dry matter MJ^−1^) based approach (Monteith, 1990; Monteith and Moss, 1977) is used to simulate crop and grass growth within the landuse module. The rate of biomass production depends on the incoming solar radiation in terms of photosynthetically active radiation (PAR, i.e. 50% of the global radiation), crop/grass specific LUE and growth affecting factors such as moisture and nutrient stresses (Coleman et al., 2017). The biomass formed is partitioned between roots, stem, leaves and storage organs based on the development stage (DVS) as described by Wolf (2012). In principle, crop phenology is expressed in terms of crop development stage (DVS), which is a function of temperature sum or growing degree days and includes the effect of vernalisation and/or photosensitivity of the crop (deVries, 1989), which are variety specific and may vary across the country. As the model works on a monthly timestep, and the flowering and maturity of the crop falls within a given month for a given crop across the whole country, we used a simple growth function to represent the DVS for each crop. We calculated DVS for each crop by applying the Landscape model for Rothamsted site for several years (1968–2012) and generated a general growth curve for each crop (Fig. 2). For grass, we assume the plant remains in vegetative phase (DVS < 1) throughout its growing period because it is continuously grazed or cut with sufficient frequency.

Crop specific parameters for different crops were taken from the model database (http://models.pps.wur.nl/glossary/l). For older varieties of wheat (developed before 1970), a few parameters were changed by calibration (see the Supplementary information (SI 5) for more details). Insufficient water and nutrients (N & P) lead to stress that affects crop growth and reduces the biomass production and yield as described by Coleman et al. (2017). The crop is harvested at maturity (when DVS = 2) and the crop yield is the weight of storage organ (g m^−2^, which could be grains, seeds or tubers). Straw or trash yield depends on the stem biomass and the method of harvesting. During the historical period (prior to 1950), we assume that crops were harvested manually and were left with less residue (15% of stem biomass) compared to the current period when machines were used (30% of stem biomass). The grass model differs from the crop only in the assumption that grass is perennial in growth and is managed differently by allowing livestock grazing or frequent cutting.

A reasonably good agreement in the model results to the measurements from Broadbalk and Park Grass (see SI 5 for model calibration and testing) for plant yield, SOC, total N and NO_3_N leaching over the last 160 years with a relative RMSE of 4–71% (see SI, Tables S5.2 and S5.3) provides confidence in Roth-CNP's ability to estimate crop and grass yields, SOC and SON for the historical to current period of 1800–2010.

### 2.2 Model inputs

Input data driving the model come from multiple sources and are described in the following sections. Some of these driving data come from readily available datasets (weather and soil). Other inputs include time-dependent datasets that have been created specifically for the LTLS-IM (landcover, landuse, livestock population, atmospheric deposition, fertilizer and manure input rates) and outputs from the loosely coupled models used either to initialise Roth-CNP (N14CP (Davies et al., 2016; Tipping et al., 2012; Tipping et al., 2017): provides initial states for soil C, N and P) or to provide dynamically-changing variables (the hydrological model provides actual evapotranspiration, soil moisture, erosion and runoff).

#### 2.2.1 Atmospheric deposition

Atmospheric N (NH_4_-N and NO_3_-N) input to arable and grassland systems were estimated for different time slices: 1800, 1900, 1950, 1970, 1990 and 2010 (see SI 1) at a 5 km × 5 km grid resolution across the UK, using land-cover dependent deposition velocities. Nitrogen deposition values for each land cover type in each grid square were interpolated for the whole period within these time slices to calculate the annual deposition rates and were averaged to calculate the monthly input rates. Nitrogen deposited in the forms of NH_4_-N and NO_3_-N was added directly to the mineral NH_4_-N and NO_3_-N pools, respectively. Atmospheric P deposition is ignored in this study as P deposition is insignificant in the UK as most of the atmospherically transported P is natural in origin (ocean, tropical forests, peatland, dust from deserts) and are redeposited to its origin (Tipping et al., 2014).

#### 2.2.2 Weather

For weather, data from several sources were combined to derive an observation-based dataset from 1800 to present. For rainfall (mm month^−1^), daily observations, which are available back to the 19th century, were used, although network coverage ranges from only 2 rain gauges in 1853 to thousands in the late 20th century. National daily rainfall estimates for each a 5 km × 5 km UK grid square were derived from any daily observations available for the period 1853 to 1910. From 1910 onwards, gridded 1 km resolution rainfall observations from CEH-GEAR (doi:https://doi.org/10.5285/5dc179dc-f692-49ba-9326-a6893a503f6e) were used. No observed daily rainfall values were available prior to 1853, so daily rainfall for a median year (1904) was assumed to be representative for the period from 1800 to 1853.

For all other weather variables (temperature (°C), shortwave radiation (W m^−2^), wind speed (m s^−1^) surface pressure (Pa) and specific humidity (kg kg^−1^), monthly mean values were used. These were obtained from the WATCH forcing dataset (http://www.eu-watch.org/) for the period 1800 to 1970 (data for the “median” year 1904 was assumed to be representative for the pre-1901 period), and similar data from the UK Met Office (http://www.metoffice.gov.uk) were obtained for the later period 1970 to 2010. The Met Office dataset provided observation-based estimates of minimum and maximum temperature (°C), sunshine hours (h), wind speed (m s^−1^) and vapour pressure (hPa). Prior to 1901, these data were not available for the whole country, and again, data from a nominal year (1904) is used. Note that although the WATCH forcing dataset provides weather estimates from 1901 to 2001, prior to 1958 these consist of statistically re-ordered data from 1958 to 2001, and as such do not provide a historically accurate weather record for the earlier period. In the UK, only rainfall data are available at a daily time-step with national coverage before 1958 (CEH-GEAR) and these have been used in preference to WATCH rainfall estimates. Any inconsistency incurred through the use of CEH-GEAR rainfall is assumed to be small compared to the use of the statistically correct but historically-inaccurate WATCH rainfall series. Short wave radiation in W m^−2^ was converted to MJ m^−2^. Surface pressure and specific humidity were used to calculate vapour pressure (KPa) (Nievinski, 2009).

#### 2.2.3 Land cover and landuse

A land cover history for the UK was constructed using contemporary landuse datasets and the few historical maps available (see SI 2). Livestock populations and agricultural landuse data were estimated for four time slices: 1900, 1950, 1970 and 1990 and assumed constant between these dates. Five major crops (winter wheat, spring barley, oil seed rape, potato and fodder maize) were selected, which represented five major groups of crops (winter cereals, spring cereals, Oil seed crops, tuber crops and fodder crops) in the UK. The area under each of these crops represented the sum of the total area of all the crops within each of these groups. For example, the area under winter wheat represented the total area under winter wheat, winter barley and winter oats. Similarly, spring barley represented the area under both spring barley and spring wheat. Area under potato represented the area under potato and sugar beet and all the fodder crops under fodder maize. Livestock classes considered were beef, dairy, sheep, pig and poultry. Estimates were based on historic agricultural census data and were distributed using the AENEID model (Dragosits et al., 1998; Hellsten et al., 2008) explained in SI3 with the land cover data summarised in SI 2.

The arable area in each grid cell was assumed to grow up to a maximum of five representative major crops: winter wheat, spring barley, potato, oil seed rape, and fodder maize depending on their presence or absence in that grid cell.

Under improved grass (i.e. anthropogenically managed grassland), four types of grass land management: dairy, beef, sheep and silage (ungrazed) were simulated according to the livestock population at that location.

To estimate the area under each of these livestock management systems (*A*_*i*_), we used the livestock numbers in each grid (*N*_*i*_) and the standard stocking rate (*D*_*i*_, animals/ha) for different species of livestock

(2) $$A_{i\;=\;\frac{N_{i}}{D_{i}}}$$

where *i* represents livestock species such as dairy, beef and sheep.

Stocking rates may have been different in the past especially when the livestock population was much lower than today. Due to lack of any such information for the past, we use the current standard stocking rates which are 2 (dairy), 3.3 (beef) and 20 (sheep) (Nix, 2003) for the entire study period. Any grass areas left after allocating to different livestock management were assumed to be ungrazed (hay or silage). In locations where the grass area was smaller than that estimated based on the livestock population, the model stocking rate was increased to achieve the observed population.

After 1950, further expansion of agriculture occurred with more of the semi-natural land converted to improved grass and improved grass converted to arable whilst a modest area of arable land became improved grass.

#### 2.2.4 Soil

Soil texture and soil profile depth maps for 5 km × 5 km grid cells required by Roth-CNP were created from the Harmonised World Soil Database (HWSD) (http://webarchive.iiasa.ac.at/Research/LUC/External-World-soil-database/HTML/). Soil organic C, N, P and mineral P pools were initialised with the outputs from semi natural model, N14CP (Davies et al., 2016; Tipping et al., 2012) at the point of their transition to agriculture on 1800 and 1950 (See SI, Fig. S2.1). The N14CP model assumes three SOM pools (fast, slow and passive) to describe SOC, N and P dynamics compared to four active pools within the Roth-CNP.

Total SOC from N14CP was distributed between Roth-CNP's carbon pools for both surface and subsurface layers according to the RothC initialisation as follows:

(3) $${\mathrm{TSOC}}_{N14\mathrm{CP}}={\mathrm{SOC}}_{\mathrm{fast}}+{\mathrm{SOC}}_{\mathrm{slow}}+{\mathrm{SOC}}_{\mathrm{passive}}$$

$${\mathrm{DPM}}_{C}={\mathrm{TSOC}}_{N14\mathrm{CP}}\times 0.1$$

$${\mathrm{RPM}}_{C}={\mathrm{TSOC}}_{N14\mathrm{CP}}\times 0.13$$

$${\mathrm{BIO}}_{C}={\mathrm{TSOC}}_{N14\mathrm{CP}}\times 0.02$$

$${\mathrm{HUM}}_{C}={\mathrm{TSOC}}_{N14\mathrm{CP}}\times 0.75$$

Here *TSOC*_*N*14*CP*_, *SOC*_*fast*_, *SOC*_*slow*_, *SOC*_*passive*_ refer to the total, fast, slow and passive N14CP SOC pools and *DPM*_*C*_, *RPM*_*C*_, *BIO*_*C*_, *HUM*_*C*_ represent the carbon redistributed to DPM, RPM, BIO and HUM Roth-C pools (Coleman et al., 1997).

Total organic N and P were redistributed to Roth-CNP pools based on the C/N or C/P ratios of fast, slow and passive pools of N14CP model as follows:

(4) $${\mathrm{TSON}}_{N14\mathrm{CP}}={\mathrm{SON}}_{\mathrm{fast}}+{\mathrm{SON}}_{\mathrm{slow}}+{\mathrm{SON}}_{\mathrm{passive}}$$

$${\mathrm{DPM}}_{N}={\mathrm{DPM}}_{C}/{\mathrm{CN}}_{\mathrm{fast}}$$

$${\mathrm{RPM}}_{N}={\mathrm{RPM}}_{C}/{\mathrm{CN}}_{\mathrm{slow}}$$

$${\mathrm{BIO}}_{N}={\mathrm{BIO}}_{C}/{\mathrm{CN}}_{\mathrm{BIO}}$$

$${\mathrm{HUM}}_{N}={\mathrm{TSON}}_{N14\mathrm{CP}}-\left({\mathrm{DPM}}_{N}+{\mathrm{RPM}}_{N}+{\mathrm{BIO}}_{N}\right)$$

The BIO pool of Roth-CNP is largely microbial in nature and is assumed to have a fixed C/N (8.5) and C/P (50) ratios. In a similar way, SOP was also allocated to different Roth-C pools. In this way, fast and slow pools C, N and P from N14CP were allocated to the corresponding pools within the Roth-CNP model, without creating or losing C, N and P.

#### 2.2.5 Hydrology

Hydrological inputs such as AET (mm month^−1^), soil moisture (mm), drainage (mm month^−1^) and runoff (mm month^−1^) on a 5 × 5 km square grid covering the UK were estimated by the hydrology component of the LTLS-IM (Bell et al., in prep). The hydrology model is summarised in Supplementary information (SI 4).

#### 2.2.6 Fertilizer

**Fig. 3:**
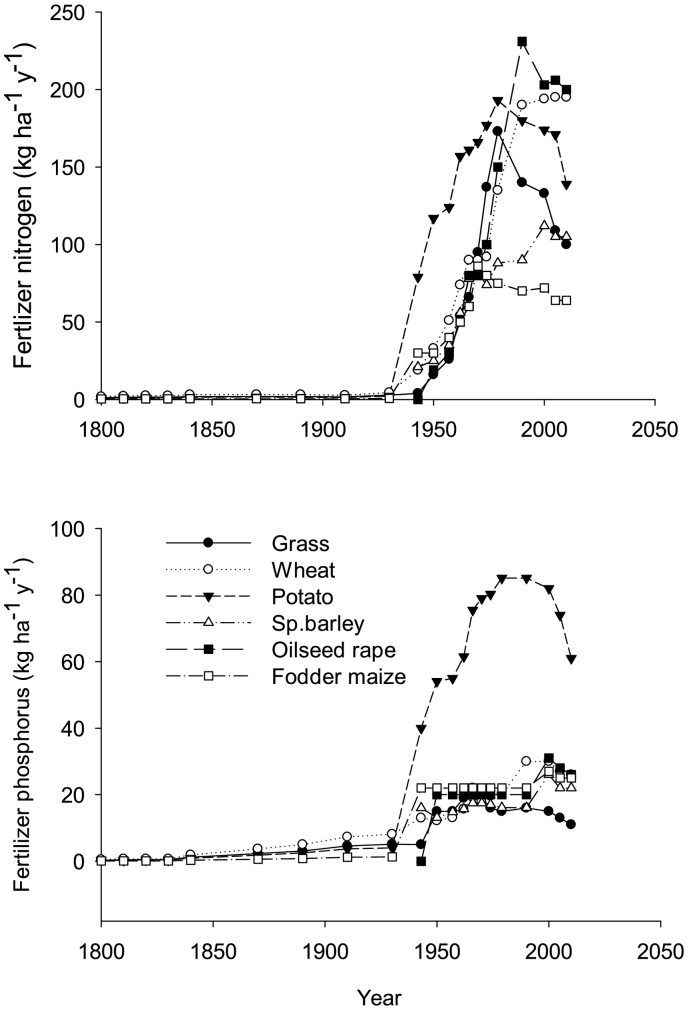
Historical to current rates of nitrogen and phosphorus fertilizer application rates under grass and crops (Archer, 1985; DEFRA, 2011b; Naden et al., 2016).

Manure and fertilizer application rates to arable and grass land were calculated based on the information available from various sources. During the period 1800 to 1840s, sewage in the form of “night soil” was applied to crops and grass (Naden et al., 2016). After 1840, imported N fertilizers (seabird guano, Chilean nitrate) and superphosphate were applied in small amounts. Average N fertilizer input to agricultural land during this period was calculated based on the total fertilizer use (Archer, 1985) and the total area under agriculture (see Section 2.2.3). The average per hectare fertilizer use increased from 7.2 to 13.1 kg ha^−1^ for N and 4.4 to 16.2 kg ha^−1^ for P during 1840 to 1940 (Archer, 1985), with 75% of these nutrients were assumed to be applied to arable and 25% to the grass. Chemical fertilizers were applied from 1940s and their rates increased over the years (Archer, 1985; DEFRA, 2011b). For example, N fertilizer application in winter wheat increased from 19 to 195 kg N ha^−1^ and 4 to 100 kg N ha^−1^ for grass during 1943 to 2010 (Fig. 3). Mineral N fertilizer application before 1940 was small.

#### 2.2.7 Manure

Manure contribution by deposition of grazing animals (beef, dairy and sheep), slurry, and poultry is calculated based on livestock population and their daily manure (dung and urine) excretion rate.

Carbon and nutrient contributions from deposition of grazing animals depend on the frequency of manure deposition, dry matter (DM) content, carbon, organic-N, NH4-N, and P content of the urine and dung for different livestock species (Table 1). Carbon and nutrient concentrations in dry matter are estimated by the equations

(5) $$C_{\mathrm{dep},j,k}=F_{\mathrm{dep},j,k}\;\;{\mathrm{eC}}_{\mathrm{dep},j,k}$$

(6) $$N_{\mathrm{dep},i,j,k}=F_{\mathrm{dep},j,k}\;\;{\mathrm{eN}}_{\mathrm{dep},i,j,k}$$

where *C*_*dep*, *j*, *k*_ and *eC*_*dep*, *j*, *k*_, are the carbon content (g) and carbon concentration in the dry matter (g event^−1^). *F*_*dep*, *j*, *k*_ is the frequency of occurrence of dung or urine event (month^−1^) for different animal species. *N*_*dep*, *i*, *j*, *k*_, is the nutrient, *i* (NH_4_-N, NO_3_-N, organic N, inorganic P and organic P) deposited (g animal^−1^ month^−1^) in the form of dung or urine (*j*) of different livestock species (*k*) and *eN*_*dep*, *i*, *j*, *k*_ is the nutrient deposited by urine or dung event (g event^−1^).

**Table 1:** Parameters used to calculate the carbon and nutrient contribution from manures.


Parameters	Dairy	Beef	Sheep	Pig	Poultry	Reference
Manure (dung and urine) deposition						
Frequency of deposition of dung, (month^−1^)	360	300	660	−	−	(Lantinga et al., 1987; McGechan and Topp, 2004; Orr et al., 2014; Williams and Haynes, 1995)
Frequency of deposition of urine, (month^−1^)	360	258	510	−	−	(Lantinga et al., 1987; McGechan and Topp, 2004; Rosen et al., 2004; Wheeler, 1959)
Carbon deposited, _(_g C event^−1^)	90	106	14	−	−	(Orr et al., 2014; Whitehead, 1995; Williams and Haynes, 1995)
Organic-N deposited, _(_g C event^−1^)	0.32	0.83	0.19	−	−	Whitehead (1995)
Organic-N deposited, (g N event^−1^)	1.07	3.88	0.55	−	−	(Lantinga et al., 1987; Sakadevan et al., 1993)
NH_4_-N deposited, (g N event^−1^)	0.01	0.03	0.003	−	−	(Sakadevan et al., 1993; Whitehead, 1995)
NH_4_-N deposited, _(_g N event^−1^)	6.07	11.07	0.05	−	−	(Lantinga et al., 1987; Sakadevan et al., 1993; Whitehead, 1995)
Total-P deposited, _(_g P event^−1^)	1.40	0.01	0.21	−	−	(Haynes and Williams, 1993; Orr et al., 2014; Williams and Haynes, 1995)
Total-P deposited, _(_g P event^−1^)	0.00	1.20	0.00	−	−	(Manston and Vagg, 2009; Orr et al., 2014; Shand et al., 2002)

Slurry						
Volume, (m^3^ month^−1^)	1.5	0.90	−	0.15	−	DEFRA (2011a)
Volume fraction of dry matter, (m^3^ m^−3^)	0.06	0.06	−	0.04	−	DEFRA (2010)
Density, (g m^−3^)	1,040,000	1,040,000	−	800,000	−	
Carbon concentration, (g C g^−1^ DM)	0.20	0.20	−	0.2	−	MAFF (1998)
Organic-N concentration, (g N m^−3^)	1900	2300	−	1300	−	ADAS (2007)
NH_4_ = N concentration, (g N m^−3^)	1300	2000	−	2300	−	ADAS (2007)
Total-P concentration, (g P m^−3^)	622	933	−	0.025	−	ADAS (2007)

Poultry manure						
Dry matter, (g DM month^−1^)	−	−	−	−	2.5	DEFRA (2011a)
Carbon concentration, (g C g^−1^ DM)	−	−	−	−	0.24	MAFF (1998)
Total-N, (g N month^−1^)	−	−	−	−	0.048	Nicholson et al. (1996)
Total-P, (g P g^−1^ DM)	−	−	−	−	0.015	Nicholson et al. (1996)

Slurry is collected when cattle are housed during winter (for dairy and beef). Slurry production depends on the slurry volume, density, DM content, and the nutrient content (Table 1) of livestock species (beef, dairy and pig) as follows:

(7) $$C_{\mathrm{sl},i,k}=V_{\mathrm{sl},k}\;{\mathrm{fDM}}_{\mathrm{sl},i,k}\;D_{\mathrm{sl},i,k}\;{\mathrm{cC}}_{\mathrm{sl},i,k}$$

(8) $$N_{\mathrm{sl},i,k}=V_{\mathrm{sl},k}\;{\mathrm{vN}}_{\mathrm{sl},i,k}\;$$

where *C*_*sl*, *i*, *k*_ is the carbon (g C animal^−1^ month^−1^) in the slurry of livestock species *k*. The variables *V*_*sl*, *k*_ , *fDM*_*sl*, *i*, *k*_ and *D*_*sl*, *k*_ represent the volume (m^3^ month^−1^), volume fraction of DM (m3 m^−3^) and density (g m^−3^) of the slurry collected from each animal for a given livestock species *k*, and *cN*_*sl*, *i*, *k*_ is the nutrient concentration (g nutrient kg^−1^ of DM) of the slurry for a given livestock species *k*.

Poultry manure is collected during the whole year and of rate of C (*C*_*man*, *k*_) and nutrients (*N*_*man*, *i*, *k*_) produced (g animal^−1^ month^−1^) is given by

(9) $$C_{\mathrm{man},k}={\mathrm{DM}}_{\mathrm{man},k}\;{\mathrm{cC}}_{\mathrm{man},k}$$

(10) $$N_{\mathrm{man},i,k}={\mathrm{DM}}_{\mathrm{man},i,k}\;{\mathrm{cN}}_{\mathrm{man},i,k}\;$$

depends on the manure DM production (*DM*_*man*, *k*_, g DM month^−1^) and C (*cC*_*man*, *i*, *k*_, g C g^−1^ DM) and nutrient concentration ( *cN*_*man*, *i*, *k*_,g nutrient g^−1^ DM) (Table 1).

Nutrient input from animal excreta is not directly linked to the nutrient concentration of the grass the animals eat because it is likely that animals also eat food supplements that could affect the nutrient concentration in their excreta. A part of NH_4_-N is lost through volatilization (from manure management practice (housing, manure storage & application to land) and is found to be 0.09 and 0.60 for NH_4_-N in the urine and dung deposition by cattle and sheep, respectively (McGechan and Topp, 2004; Whitehead, 1995). For slurry, volatilization fractions for dairy, beef and sheep are 0.6, 0.31 and 0.6, respectively. For poultry manure the volatilization loss fraction is 0.3.

### 2.3 Historical to current simulation

Prior to 1800, landuse in the UK was largely semi-natural, dominated by natural forests and low input agriculture. Before 1800, a semi-natural model (N14CP, Davies et al., 2016) was used to simulate all landcover including agriculture and to estimate the carbon stocks and nutrient fluxes from 10,000 BP onwards. Following the agricultural revolution (assumed to take place in 1800), Roth-CNP was used to provide a dedicated simulation of agricultural practices in arable and improved grasslands while N14CP continued to simulate nutrient pools and fluxes in semi-natural areas. The soil macronutrient initial condition required by Roth-CNP to allow simulations to start at 1800 was provided by N14CP. A further expansion of agriculture into previously semi-natural areas in the mid-20th Century, assumed to take place in 1950, necessitated a second exchange of soil nutrient pools between N14CP and Roth-CNP in these areas.

**Fig. 4:**
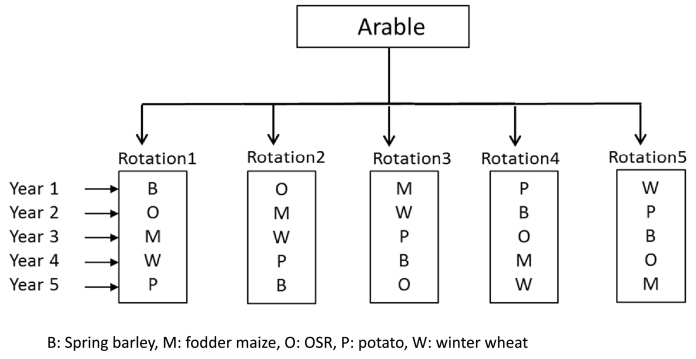
An example scheme of crop rotation in a grid cell with five crops. (This results in five crop rotations with five crops in each individual rotation on a five-year cycle. This scheme will be adapted when the number of crops in a grid cell is less than five by reducing the number of crop rotations, number of crops in each rotation and the duration of the crop rotation cycle).

Here we aim to estimate C, N and P pools, pool changes, their balance and the nutrient fluxes exported from arable and grassland systems in the UK on a 5 × 5 km grid across the whole of UK during the historical to current period (1800 to 2010). Crop and grass landuse models were run separately on the arable and grassland area within each 5 km grid cell. Based on the five crops in landuse (Section 2.2.3), we identified a maximum of five possible crop rotations with the actual number of rotations dependent on the number of crops in each grid cell (Fig. 4). The number of simulations in each grid cell depends on the number of these rotations and the model variables are re-initialised each time during these simulations. In this way, all the crops that are present in each grid cell are simulated in each year. To calculate the mean yield of a crop we took the weighted average of the yield for each crop for each year by multiplying the area of the crop in each rotation at the end of all the simulations for all the rotations.

Similarly to crop rotations, the model runs for different grazing management systems after re-initialising the model variables for soil and plant growth at each time and the nutrient fluxes are calculated as weighted averages of the area under each grazing management.

Under different livestock management systems, animals graze from April to September and the rate of manure (urine and dung) input and the grass removed depends on the stocking rate and animal species (Coleman et al., 2017). During winter when animals are housed, the manure is collected, stored and applied in March in the form of slurry. Nitrogen and P fertilizers are also applied and their rate increases over time, peaking in the late 20th century before starting to decline in more recent years (Fig. 3). All of the P fertilizer is applied in spring whereas N fertilizer is applied in splits (up to 6 in 1990 compared to one single application in 1950 (DEFRA, 2010).

**Fig. 5:**
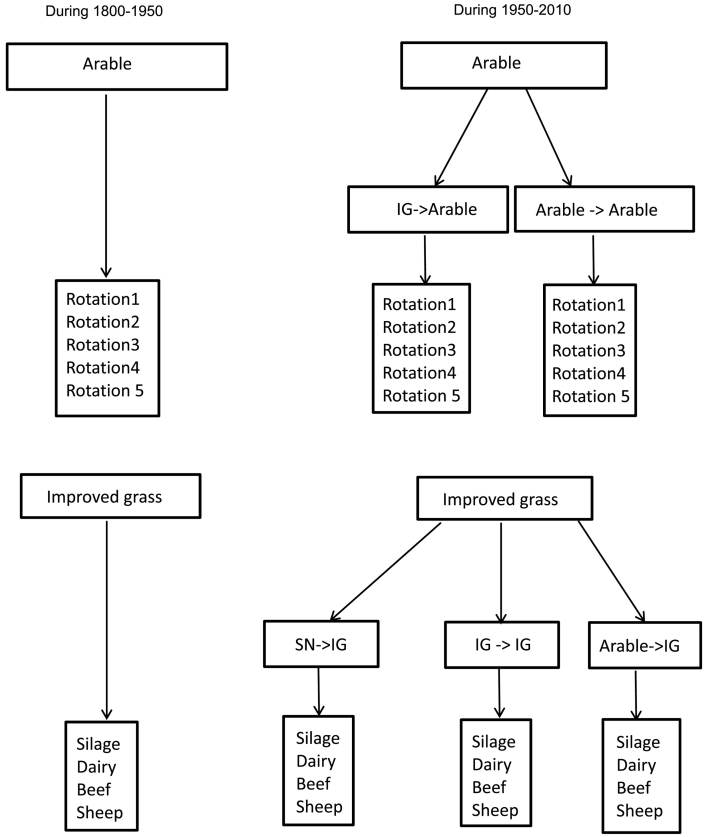
Schematic highlighting the different crop rotation and livestock simulation schemes used by Roth-CNP to simulate arable and improved grassland histories (SN: semi-natural; IG: improved grass) before and after land cover changes applied in 1950.

In 1950, widespread landcover change in the UK resulted in different landcover histories depending on the location (grid cell) (Fig. 5). For computational simplicity, Roth-CNP soil variables were reinitialised in 1950 with the outputs from the semi-natural model N14CP (Davies et al., 2016) to incorporate new landcover histories applied from 1950 onwards.

Simulated model results were analysed in three different periods: *historical* (1800–1950), *transition* (1950–1970) and *current* (1970–2010), which are distinct in terms of landuse and agronomic practices. During the historical period, agriculture was more traditional with local varieties and manure and/or slurry based fertilizer inputs. During the transition (post war) period, widespread land cover changes occurred alongside increased use of chemical nitrogen fertilizers in agriculture. The current period is characterised by the so called ‘green revolution’ effect where improved crop varieties, mechanisation, increased livestock population with higher inputs of chemical fertilizers and pesticides were used, supplementing but also disturbing the natural cycle of C, N and P (Galloway et al., 2004). To calculate the average of a nutrient variable (e.g.: C input, NO_3_-N leached) in each grid cell, we calculated the weighted average for each variable for each year by multiplying with the area under arable or grass land in the cell. The overall C, N and P balance for the whole of UK was calculated by averaging the mean values for these different variables for different time periods across all the grid cells.

In summary, the changes of SOC (*∆SOC*, kg N ha^−1^ y^−1^), mineral N (*∆N*, kg N ha^−1^ y^−1^) and P (*∆P*, kg P ha^−1^ y^−1^) averaged for the whole of UK are then calculated as

(11) $$∆\mathrm{SOC}=C_{\mathrm{plant}}+C_{\mathrm{animal}}-{\mathrm{CO}}_{2,\mathrm{loss}}-{\mathrm{DOC}}_{\mathrm{loss}}-{\mathrm{POC}}_{\mathrm{loss}}$$

(12) $$∆N=N_{\mathrm{dep}}+N_{\min}+N_{\mathrm{BNF}}+N_{\mathrm{fert}}-N_{\mathrm{loss}}-N_{\mathrm{denit}}-N_{\mathrm{uptk}}$$

(13) $$∆P=P_{\min}+P_{\mathrm{fert}}-P_{\mathrm{loss}}-P_{\mathrm{uptk}}$$

where *C*_*plant*_ and *C*_*animal*_ are the overall mean average annual carbon input through plant and animal sources (Mg C ha^−1^ y^−1^), *CO*_2, *loss*_ is the loss (Mg C ha^−1^ y^−1^) of SOC in the form of CO_2_ through microbial respiration, *DOC*_*loss*_ is the loss of SOC (Mg C ha^−1^ y^−1^) in the dissolved form through leaching and runoff, and *POC*_*loss*_ is the loss through soil erosion in the particulate form. *N*_*dep*_, *N*_*min*_, *N*_*BNF*_,  *N*_*fert*_ are the overall N inputs through atmospheric deposition, SOM mineralisation/immobilisation, biological N fixation and fertilizer N application (all in kg N ha^−1^ y^−1^). *N*_*loss*_,  *N*_*denit*_ and *N*_*uptk*_ are loss of nitrogen through leaching, runoff and soil erosion and N removed from soil by plant uptake (all in kg N ha^−1^ y^−1^). *P*_*min*_ and *P*_*fert*_ are the overall P inputs through SOM mineralisation and fertilizer P application and *P*_*loss*_ and *P*_*uptk*_ are loss the of P through leaching, runoff and soil erosion and P removed from soil by plant uptake (all in kg P ha^−1^ y^−1^).

In the model, nutrient (N and P) inputs through litter and those removed by plant uptake are calculated separately. Plant nutrient (N and P) uptake is the cumulative uptake during the whole growing season and a part of these nutrients goes back to soil when plant residues are returned after the harvest (by keeping track of the nutrient concentration in different organs). Litter enters soil SOM pools and undergoes decomposition and forms NH_4_-N by mineralisation and forms NO_3_-N by nitrification.

## 3 Results

### 3.1 Historical to current simulation

#### 3.1.1 Historical period (1800–1950)

**Fig. 6:**
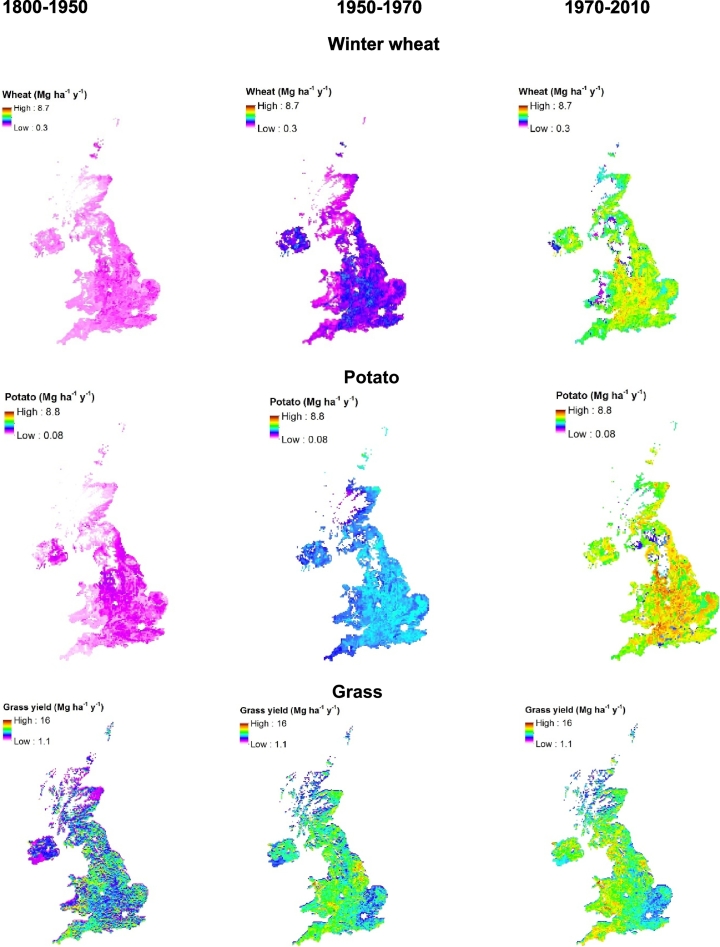
Simulated average wheat, potato and Grass (grazed and/or cut) yields (Mg DM ha^−1^) at different time periods (1800–1950, 1950–1970 and 1970–2010) across the whole UK.

Simulated wheat yields ranged from 0.3 to 1.9 Mg DM ha^−1^ with an overall mean average yield of 1.0 Mg ha^−1^ (Fig. 6; Table 2). Simulated potato yields were similar to those of wheat and ranged from 0.1 to 2.0 Mg DM ha^−1^ with an overall mean average yield of 0.9 Mg ha^−1^ and simulated fodder maize yield had an overall mean average yield of 4.9 Mg DM ha^−1^. For both wheat and potato, simulated yields ranged between 0.08 and 2.0 Mg ha^−1^ (Fig. 6) lower than that reported for this period in national statistics (Table 2). Simulated grass yields varied widely across the UK from 1.3 to 16 Mg ha^−1^ (Fig. 6), with the lowest yields occurring mostly in Northern Scotland and Northern Ireland, where SOC was lower than elsewhere. A lower SOC indicates lower SON and SOP and lesser availability of N and P for plant uptake through their mineralisation.

**Table 2:** Overall mean average simulated crop/grass yields (Mg dry matter ha^−1^) compared to that reported by national statistics^a^ for different time periods in the UK.

Crop/grass	1800–1950		1951–1970		1971–1990		1991–2010	
	Simulated	Reported	Simulated	Reported	Simulated	Reported	Simulated	Reported
Winter wheat	1.0	1.9^b^	2.4	3.1	4.8	4.8	6.1	6.5
Potato	0.9	3.2^b^	3.4	4.4	5.9	6.5	6.1	8.2
Spring barley	−	−	−	−	3.9	4.1	4.2	4.8
Oilseed rape	−	−	1.6	NA	1.9	2.5	3.0	2.8
Fodder maize	4.9	NA	7.4	NA	7.6	NA	6.9	NA
Grass	6.8	NA	7.9	NA	8.8	NA	9.2	NA

**Fig. 7:**
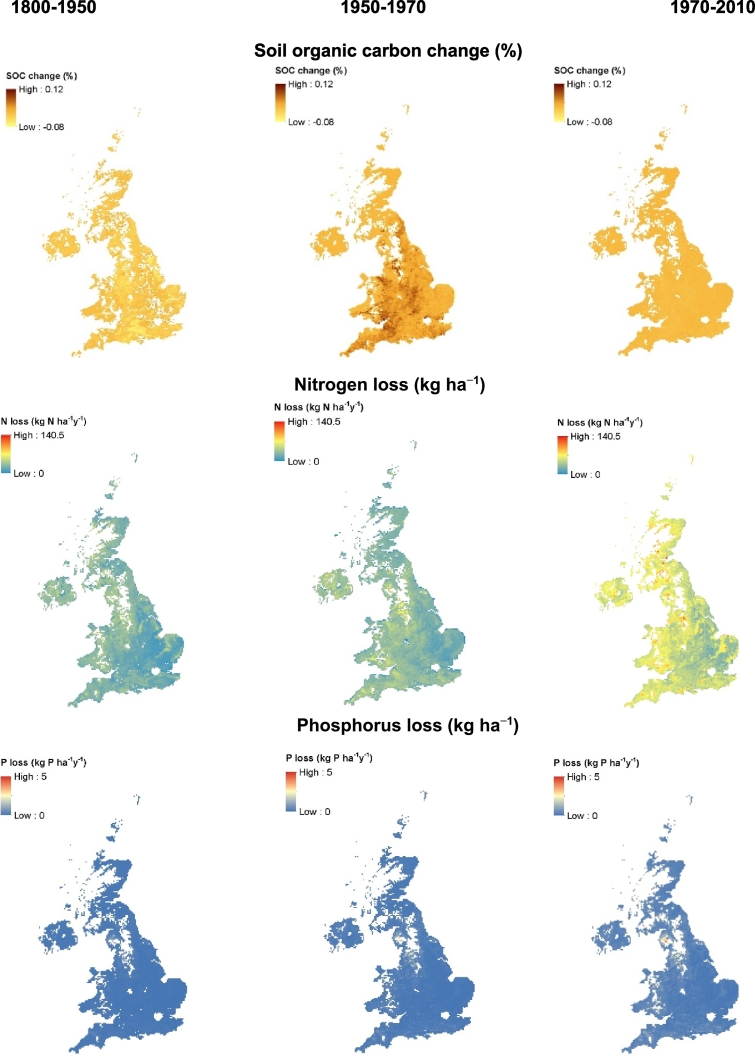
Simulated soil organic carbon change, average annual N and P losses (leaching + runoff) at different time periods (1800–1950, 1950–1970, and 1990–2010) under arable land for the whole UK.

**Fig. 8:**
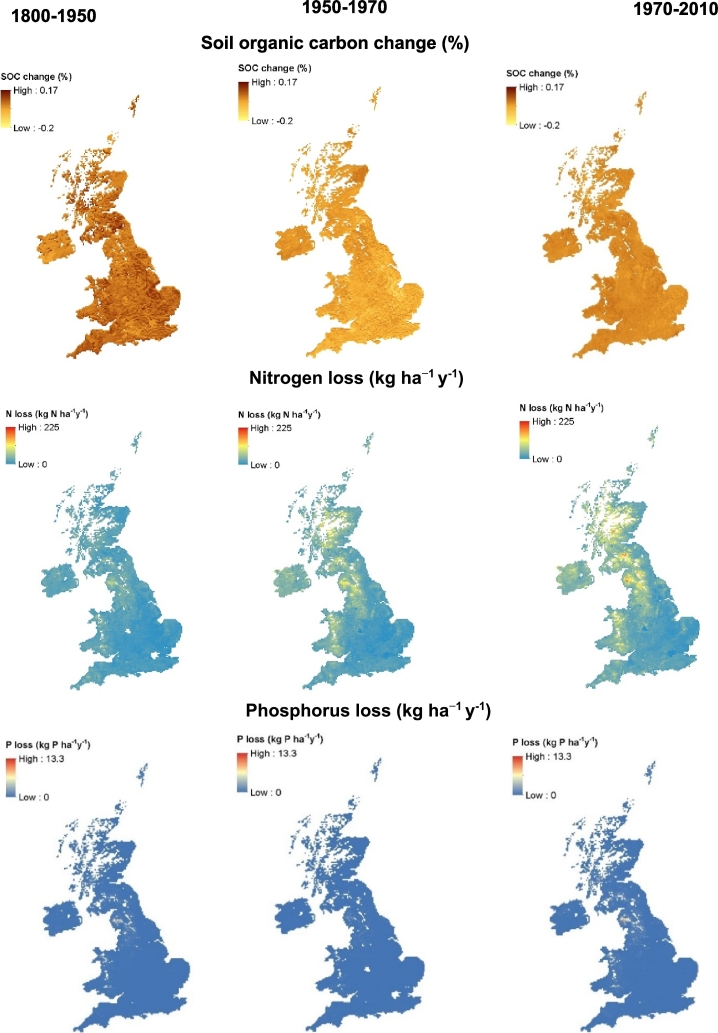
Simulated soil organic carbon change (%), average annual N and P losses at different time periods (1800–1950, 1950–1970 and 1970–2010) under grass land for the whole UK.

NA: Not available.

a MAFF (1988); Marks and Britton (1989).

b 1884–1950;

For arable land, simulated average annual SOC change during the historical period is small (−0.08 to 0.12%) (Fig. 7) across whole of the UK with an overall mean net carbon change of −0.18 Mg C ha^−1^ y^−1^ (Table 3). During the same period, there was a general build-up of simulated SOC with an overall mean net carbon change of 0.2 Mg C ha^−1^ y^−1^ under grass land with a change in carbon ranging from −0.2 to 0.17% annually (Fig. 8). Simulated plant and animal C input to the grass land was greater (2.9 Mg C ha^−1^ y^−1^) compared to that under arable (1.0 Mg C ha^−1^ y^−1^). About 93% of simulated total (plant plus animal) carbon input under grass was decomposed, resulting in the build-up of C by 0.2 Mg C ha^−1^ y^−1^.

**Table 3:** Overall mean average annual soil carbon, nitrogen and phosphorus balance^a^ (for the whole profile) for arable and grass lands estimated based on simulation results for different time periods for whole of the UK.

Components	1800–1950		1950–1970		1970–2010	
	Arable	Grass	Arable	Grass	Arable	Grass
Soil organic carbon (Mg ha^−1^ y^−1^)						
Plant C input	1.01	2.88	0.99	3.40	1.05	3.86
Animal C input	0.01	0.70	0.02	0.65	0.03	0.75
Dissolved organic carbon loss	0.00	−0.04	0.00	−0.04	0.00	−0.04
Particulate organic carbon loss	0.00	−0.01	0.00	−0.01	0.00	−0.02
Carbon loss (by decomposition as CO_2_)	−1.2	−3.33	−1.25	−3.53	−1.16	−4.30
Net carbon change	−0.18	0.20	−0.25	0.47	−0.08	0.25

Mineral nitrogen (kg ha^−1^ y^−1^)						
Atmospheric N deposition	3.9	4.03	8.8	9.09	11.5	11.91
Fertilizer N input	8.1	2.03	64.0	35.0	127.9	134.8
N input by mineralisation	38.7	67.3	41.0	65.92	41.4	81.73
Animal N input	0.9	43.2	2.1	41.44	2.9	48.53
N input by biological N fixation	0.0	47.4	0.0	54.30	0	43.4
N loss by leaching, runoff and soil erosion	−14.9	−17.7	−29.0	−21.47	−52.3	−36.02
N loss by denitrification	−0.3	−0.28	−0.78	−0.38	−1.49	−0.61
Plant N uptake	−35.9	−144.9	−79.3	−173.9	−128.9	−283.6
Net N change	0.50	0.98	6.8	10.0	1.0	0.14

Mineral phosphorus (kg ha^−1^ y^−1^)						
Fertilizer P input	8.7	2.34	26.4	16.67	34.6	14.54
P input by mineralisation	5.6	10.2	6.6	9.19	5.41	11.62
Animal P input	0.10	8.0	0.2	7.75	0.32	7.93
P loss by leaching, runoff and soil erosion	−0.03	−0.03	−0.14	−0.05	−0.28	−0.14
Plant P uptake	−11.8	−17.8	−22.3	−22.25	−22.0	−30.34
Net P change	2.57	2.8	10.8	11.3	18.05	3.61

a A Positive sign indicates input or gain and negative sign indicates loss from the soil system.

For arable land, the major part of estimated N input during the historical period was from soil organic matter mineralisation (39 kg N ha^−1^y^−1^) (Table 3). Simulated N was removed from soil mainly by crop offtake (36 kg N ha^−1^ y^−1^) followed by losses through leaching, surface runoff and soil erosion. Simulated N loss varies across the country with an overall mean average of 15 kg N ha^−1^ y^−1^ (Table 3; Fig. 7). However, simulated N loss through denitrification was relatively smaller (0.3 kg N ha^−1^ y^−1^). For grassland, overall simulated total N input was about 164 kg N ha^−1^ y^−1^ with the major contribution from N mineralisation (67 kg N ha^−1^ y^−1^) and BNF (47 kg N ha^−1^ y^−1^). Simulated N loss ranged across the country with an overall mean loss of 18 kg N ha^−1^ y^−1^ (Fig. 8; Table 3). The net rate of change of N under grass was almost double of that under arable land.

Phosphorus balance takes account of similar components to the N balance except that for atmospheric deposition and BNF (Table 3). Simulated total annual P input includes P from weathering, SOM mineralisation and fertilizer application. Under both arable and grassland, simulated P offtake and P loss through leaching, runoff and soil erosion were less than the P input and resulted in a P build up in the soil at a rate of 2.6 and 2.8 kg P ha^−1^ y^−1^ (Fig. 7, Fig. 8; Table 3).

To summarise, estimated crop yields in this period were underestimated when compared to the reported yields between 1880 and 1950. SOM under arable was lost but accumulated under grassland. Mineralisation from SOM was major source of mineral N and P (and BNF in case of N in grass) and most of these nutrients were removed through crop uptake with little remaining for loss through leaching, runoff and/or soil erosion.

#### 3.1.2 Transition period (1950–1970)

Simulated wheat (0.8 to 4.0 Mg ha^−1^) and potato (1.2 to 5.2 Mg ha^−1^) yields during the transition period were greater than that under historical period with an overall mean average yield of 2.1 Mg ha^−1^ and 3.4 Mg ha^−1^, respectively (Fig. 6, Table 3). Nevertheless, these yields were less than the reported average yield for the whole UK for 1950–1970 (Table 3). Simulated overall mean average fodder maize yield (7.4 Mg ha^−1^) increased by half compared to that during the historical period. Simulated grass yield also increased during this period especially in the western parts of the country (Fig. 6) with overall mean average annual yield increasing by 16% compared to the historical period (Table 3).

In arable land, there was a marginal increase in simulated SOC stock particularly in areas of England where grass was converted to arable land in 1950 and elsewhere, SOC changes were less apparent or even decreased (Fig. 7). In grassland, there was a decrease in simulated SOC stock in large parts of England and a marginal increase in the rest of the UK (Fig. 8). Plant derived C was the major source of C input under arable and grassland (Table 3). Under arable land, simulated SOC loss by decomposition exceeds the total C input resulting in decrease in SOC stock during this period. Simulated overall mean average annual changes during this period were − 0.25 and 0.47 Mg C ha^−1^ y^−1^ under arable and grasslands, respectively.

For arable land, the major contribution of mineral N in the model is from fertilizer application followed by N input through mineralisation and atmospheric deposition (Table 3). A large part of this nitrogen is taken up by the crop (about 70%) and 25% is lost through leaching, runoff and erosion. Simulated N loss varies across the country with greatest losses occurring in the western England and Northern Ireland (Fig. 7). For grassland, simulated overall average total N input was 206 kg N ha^−1^ y^−1^ with the major contribution of N from mineralisation and BNF followed by fertilizer application (Table 3). Simulated N removal by grass offtake is about 85% of this total N and about 11% was lost through leaching, runoff and erosion. Similarly to the arable land, N loss was greater in the western parts of the country (Fig. 8).

Simulated overall average P input (33–34 kg P ha^−1^ y^−1^) and P offtake (22 kg P ha^−1^ y^−1^) under both arable and grass were very similar resulting in an overall annual P build up at a rate of 11 kg ha^−1^ y^−1^ during this period (Table 3).

This period is characterised by an increase in yields compared to the historical period, but yields are still underestimated by the model when compared to the reported values. Soil organic carbon in the model continues to be lost under arable but built up under grassland. Mineral fertilizer became the major source of N and P in arable land whereas under grassland soil mineralisation and BNF continued to be the major sources of N. Although nutrient loss by leaching, runoff and/or erosion has increased during this period, plant nutrient uptake was the major process of nutrient removal from the soil system.

#### 3.1.3 Current period (1970–2010)

Simulated crop yields increased substantially during the current period compared to the transition period (Table 2). Winter wheat yields ranged from 1.4 to 8.7 Mg ha^−1^ with maximum yields occurring in the South and South east England (Fig. 6). Simulated overall mean average wheat yield more than doubled in the first half of the current period (1970–1990) and increased further during 1990–2010 by another 30% (Table 2). Potato yields increased in some parts of the country to about 8.8 Mg ha^−1^ with an overall mean average yield of 6.0 Mg ha^−1^ during this period. Similarly, overall mean average yields for spring barley and oilseed rape has also increased whereas fodder maize yields decreased slightly. However, simulated yields for winter wheat, potato and spring barley were lower by −6%, −25% and −12% than the reported average yields for these crops for whole of the UK during these periods. Simulated grass yields increased especially in the western parts of the UK (Fig. 6; Table 2).

For arable land, simulated SOC decline during the current period was relatively small suggesting that SOC was approaching an equilibrium. The carbon inputs from plant and animal sources were marginally increased and SOC decomposition was slightly less than in the transition period (Fig. 7, Table 3). Under grassland, SOC stock continued to build up during this period with increased carbon input through plant and animal sources with a reduced of loss C through SOC decomposition. (Fig. 8; Table 3).

In both arable and grassland systems, the major contribution of simulated N was from fertilizer (Table 3) during this period with nitrogen offtake by grass more than double of that of crops. Simulated N loss by leaching, runoff and erosion increased and were greatest during this period both under arable and grass land especially in the western parts of the UK with overall mean average losses of 52 and 36 kg N ha^−1^ y^−1^ (Fig. 7, Fig. 8; Table 3).

The overall mean average annual P fertilizer application increased under arable land (35 kg P ha^−1^ y^−1^) and decreased slightly under grassland (15 kg P ha^−1^ y^−1^) during the current period compared to the transition period. As a result, simulated P continued to build up in the soil at a higher rate especially under arable land and there was an increase in overall mean average P loss by leaching, runoff and soil erosion under both arable and grass (Table 3).

Simulated crop yields in the present period were comparable to those in the historical period. This was especially true for winter wheat, the management of which is characterised by high increases in the rates of N fertilizer application (i.e. almost double under general arable and four times grassland compared to the transition period). Simulated N and P uptake and their losses increased during this period with the increases in fertilizer application. Although the rate of P fertilizer application decreased towards the end of this period under grass, the rate of uptake of P and its loss through leaching, runoff and erosion continued to increase because of the large amounts of P in soils that accumulated in the past.

## 4 Discussion

The Roth-CNP model developed from Landscape model by simplifying the processes for a monthly time-step reproduced the observed results with varying, but satisfactory degree of goodness of fit for different fertilizer treatments in Broadbalk and Park Grass LTE (See SI 5). In general, simulated wheat yields for Broadbalk and grass yields for Park Grass followed the measured trend although yields were slightly overestimated with an overall RMSE of 1.7 and 1.3 Mg ha^−1^, respectively (Table S5.3).

**Fig. 9:**
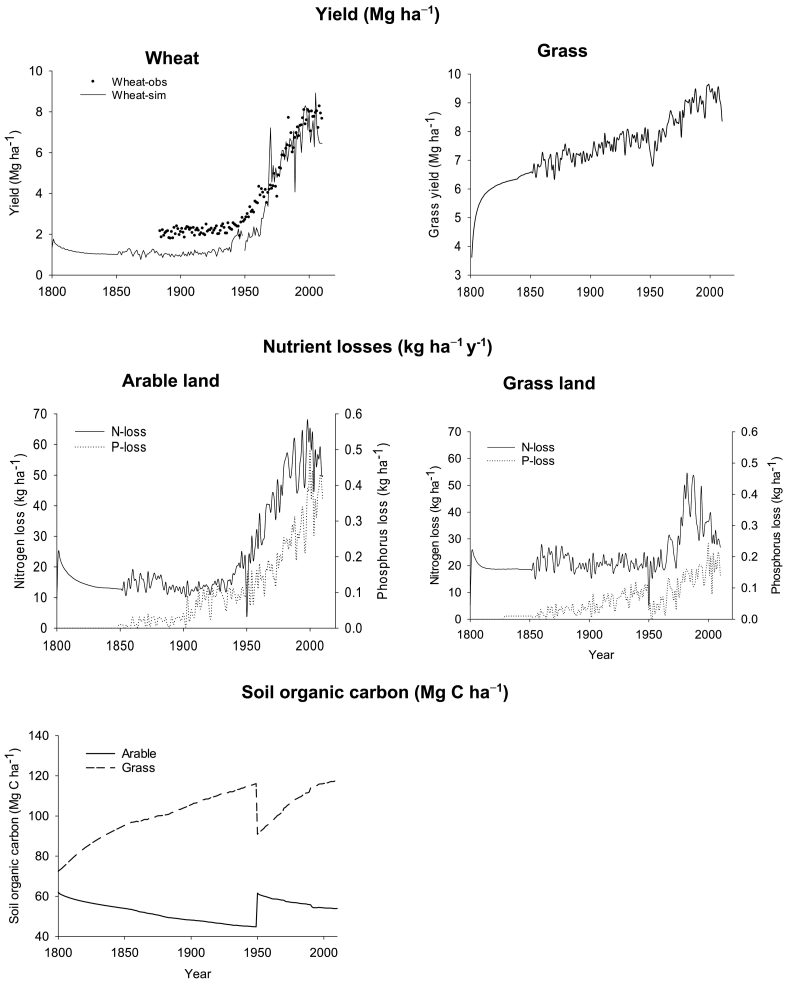
Simulated wheat yield (85% DM) compared to DEFRA reported yield statistics, simulated grass yield, nutrient losses and soil organic carbon (0–30 cm) under arable and grasslands during 1800–2010 averaged across the whole UK.

For all the crops, simulated crop yields for the whole UK show a greater yield in the north west of England for different time slices (Fig. 6). This trend is similar to the potential yield of cereals estimated by Sylvester-Bradley and Wiseman (2005) for whole UK, in which this region is characterised by greater summer rainfall and day length compared to the rest of the UK. The terrain and shallow soils may however, limit the actual production in this region. Simulated wheat and potato yields for the whole UK when compared to the national yield statistics reported by DEFRA every year since 1890s show that the model underestimated these yields during the historical and transition periods but agreed well during the current period (Fig. 9; Table 2). Availability of N for crop uptake is the major yield limiting factor in the model particularly during the historical period. This argument is based on the simulated responses of crop and grass yields to different levels of fertilizer application (please see the supplementary information, S6). Simulated winter wheat yields for Broadbalk averaged 3.5 t ha^−1^ during the historical period and 7.4 t ha^−1^ during the current period for a fertilizer application of 144 kg N ha^−1^ (Plot 8). Whereas simulated grass yields (from Park Grass) were about 7–7.5 t ha^−1^ during the historical to the current period with a fertilizer application of 96 kg N ha^−1^. Assuming a NUE of 50%, 72 kg and 48 kg N ha^−1^ will be taken up by wheat in Broadbalk plot 8 and by the grass in plot 14 of Park Grass in addition to the N contribution from mineralisation (~50 kg N ha^−1^). This could result in a nutrient uptake of about 122 kg N ha^−1^ in wheat (plot 8) and 100 kg N in grass (plot 14). Comparing that to the simulated average N uptake at the national scale (Table 3), we can see that N rates (36 kg N ha^−1^ during historical period) were not enough to achieve the potential yield of 3.5 t ha^−1^ in wheat yielding only 1 t ha^−1^ instead, whereas in grass, we can see that simulated yields are closer to the potential of 6 t ha^−1^.

Our model uses a simplified approach to development (DVS) as a function of time (Fig. 2) with detailed processes of water and nutrient dynamics to simulate the crop yields. There is a risk that a fixed DVS based on monthly instead of daily temperature will fail to capture any effect of weather or climate change on the crop duration. On balance, this should not be a problem because the effect of an increase in temperature on crop yield (a decrease of 2.5 to 10% (Hatfield et al., 2011; Lobell et al., 2011) is most likely to be offset by an increase in yield of the same magnitude of up to 11% when CO_2_ increased from 280 to 390 ppm during 1850 to 2010 (Long et al., 2005; Long et al., 2006). However, for the future simulations this may be an issue if the negative effect of increases in temperature outweigh the positive effect of an increase in yield due to increase in CO_2_ itself and vice versa.

In grass, sufficient N is taken up and a large fraction (1/3rd) of this come through BNF during the historical period. Some BNF is undoubtedly occurring in arable land too, either through leguminous crops and/or free living bacteria (Bohlool et al., 1992; Herridge et al., 2008). However, in the model we did not include either leguminous crops in the rotation or any other form of BNF, which might potentially increase modelled yields more. After 1950, simulated yield increased with the increase in fertilizer application as reported by national yield statistics, but was still underestimated. Biological N fixation might be an additional source of N. Powlson and Jenkinson (1990) reported that BNF could be contributing as much as 25 kg N ha^−1^ under fertilized treatments in Broadbalk.

An accurate comparison of the simulated and actual grass yield is not possible as the actual grass yield is removed by the livestock species and depends on grazing and grazing intensity in different parts of the country. In general, the simulated grass yields increased over the years since 1800 at an average annual growth rate of 0.6% (Fig. 9). The average grass yields (grazed or cut) estimated by the model (9 Mg ha^−1^ y^−1^) for the current period show that they are greater than the national average yield (6 Mg ha^−1^ y^−1^) (Morris et al., 2005). A greater overall mean N uptake, which is more than double that under arable system (Table 3) results in higher yields in the model. For example, during the historical period overall mean N uptake by grass is 144 kg N ha^−1^y^−1^ when N uptake in wheat is only 35 kg N ha^−1^ leading to a higher yield in the grass (6.4 t ha^−1^) compared to that under wheat (1 t ha^−1^). However, the model does not simulate any effect of changing nutrient, variety or management regimes on grass or crop quality.

Simulated SOC for whole of the UK changed in different parts of the country at different rates (Fig. 7, Fig. 8). Overall, arable lands in the UK have lost SOC during the historical to current period because carbon losses were always higher than the carbon inputs from plant residues compared to improved grasslands which gained SOC during the same period. In grassland the carbon input from plant and animal sources was greater than the loss through decomposition (Table 3). The losses of SOC (and SON and P) via erosion may be underestimated, as it is difficult to capture the effect of extreme, high intensity short duration events in erosion models, particularly at this scale. However, the simulations here provide a broad indication of the scale of C, N and P loss via erosion in comparison to other pathways. Conversely, SOC losses may be overestimated in agricultural lands on floodplains, as additions of SOC via sediment deposition during times of flood have not been considered. These are difficult to predict at the spatial and temporal scales of this approach. During the current period (1950–2010), average annual loss of SOC was at a rate of 0.22% for top soil (0–30 cm) (Fig. 9). This is similar to the SOC loss (0.38% y^−1^) reported by Reynolds et al. (2013 for England and Wales for 15 cm depth during 1978 to 2007 in their countryside survey. Under grassland, however, there was consistent build-up of SOC during this period at a rate of 0.49% y^−1^ (Fig. 9), which was higher than that reported by Reynolds et al. (2013 for improved grasslands (0.03% y^−1^). However, Bellamy et al. (2005) reported a higher loss of SOC at a rate of 0.6% y^−1^ for 15 cm depth for most soils under most landuses in England and Wales. Hopkins et al. (2009) studied the SOC trends under two long-term grassland experiments (that included Park Grass LTE used in this study to test the model) and found that there were no significant trends in SOC as these plots were showing declines, no net changes or increases in SOC. Prior to 1800, a large fraction of the grassland area simulated was under semi natural systems with relatively smaller SOC contents (see SI, Fig. S2.1) led to a build up with change in landuse from semi-natural to improved grass. A peak in SOC under arable in 1950 is due to the assumption of historical-scenario that all the landuse change which happened between 1800 and 1950 occurred in the model in 1950. As a result, a large area of improved grass land has been ploughed up then converted to arable. Similarly, a large fraction of land area changed from semi natural and arable to improved grass in 1950 resulting in a dip initially and a build-up of SOC thereafter (Fig. 9). It is quite possible that when a soil with little SOC is planted to permanent grass, SOC builds up and takes about 100 years to reach an equilibrium (Johnston et al., 2009). However, the effect of the discontinuity in the historical trend around 1950 (Fig. 9) are short-lived for components apart from SOC. A difference in the structure of the SOC model pools in N14CP and Roth-CNP may also contribute to apparent carbon build up or depletion as a result of placing some fraction of carbon from N14CP model in slow or fast decomposing pools respectively in the Roth-CNP model at the point of landuse transitions in 1800 and 1950. Further work will be needed to investigate the uncertainty in model estimates arising from the simplified historical scenarios we have assumed in this work and distribution of SOM pools between different models at their point of transfer during landuse change.

Under both arable and grass land, mineral N dynamics was dominated by different components at different time periods (Table 3). During the historical period, under arable land, the productivity was mainly determined by soil's natural fertility through N mineralisation and then fertilizers during the transition and current periods. Nitrogen mineralisation depends on SOM content and its rate of decomposition. As a result, a greater N input through mineralisation occurs under grass (65–81 kg N ha^−1^) compared to arable (39–41 kg N ha^−1^). Under grassland, BNF was always a major source of N input to soil during all periods. Legume-based N fixation can vary depending on the grass management and proportion of clover (assumed to be 30% all over in this study). The model estimated overall mean average annual N fixation to vary from 43 to 53 kg N ha^−1^ for different periods. The quantity of N fixed by high fertilizer, clover-rye grass mixture was 31–72 kg N ha^−1^ and was less than that of a low fertilizer system (120–160 kg N ha^−1^) (Høgh-Jensen and Schjoerring, 1994). There is always some uncertainty in the rates of natural biological nitrogen fixation (Galloway et al., 2004).

Simulated mineral nutrient pools and fluxes during different time periods are mainly determined by the rates of fertilizer application. Both uptake by plants and losses of nutrients increase with amounts applied. Simulated N loss by leaching, runoff and soil erosion increases through different time periods under both arable (15–52 kg N ha^−1^) and grass (18–36 kg N ha^−1^). These figures for the current period were comparable to those reported by Lord et al. (2002), who estimated N surpluses (i.e. the amount of N that could be potentially lost by leaching, runoff and denitrification) for arable and grassland were 51 kg N ha^−1^ and 23 kg N ha^−1^ (after discounting for N removal by grass) in 1995. Overall mean average N loss by denitrification in the model was negligibly small for both arable (0.3–1.5 kg N ha^−1^ y^−1^) and grassland (0.3–0.6 kg N ha^−1^ y^−1^) during different time periods. Annual denitrification is variable depending on the N-fertilizer application rate and grazing or slurry application (Whitehead, 1995). Global estimates of denitrification for different combinations soil drainage and N fertilizer application shows 10 and 14 kg N ha^−1^ y^−1^ for upland crops and grass for a fertilizer application in the range of 75–150 kg N ha^−1^ (Hofstra and Bouwman, 2005). A lower denitrification rate occurs in the model because soil is rarely saturated as the soil water is uniformly distributed in the profile as a result of averaging across the whole soil depth. This is a weakness of our approach where soil water is not integrated within the soil model and the total soil moisture storage (mm) estimated by the hydrology model is averaged for the profile depth in the soil model. Although total moisture is same, its distribution within a profile (in different soil layers) may vary depending the season: relatively more water stored at the surface layer during the autumn (rainy season) and at the lower layers during the summer (dry season). Nitrogen offtake estimated by our model for arable (128 kg N ha^−1^) and grass (284 kg N ha^−1^) were higher than that estimated for arable (100 kg N ha^−1^) and grass (116 kg N ha^−1^) land for the whole UK (Lord et al., 2002). Intensively managed grassland, which is harvested by cutting or grazing may yield between 8 and 15 Mg ha^−1^ y^−1^ of DM and contain 200–550 kg N ha^−1^ (Whitehead, 1995). In that case, for an average simulated yield of 9 Mg ha^−1^ y^−1^ (Table 2), the grass may well take up >250 kg N ha^−1^ y^−1^. A small loss estimated for denitrification may also contribute to high N uptake in the model especially under grass.

Phosphorus loss varies across the UK with maximum losses found in the North-west England where soils are shallow (Fig. 7, Fig. 8). Similarly to N, overall mean annual P loss through leaching, runoff and soil erosion increased over the years (Fig. 9) with increase in the P fertilizer application (Fig. 3). Simulated P builds-up in soil during different time periods under both arable and grassland. Withers et al., 2001 estimated the P balance for the whole of UK both under arable and grassland systems for 1993 showing that there was a surplus of 19 and 12 kg P ha^−1^. Simulated overall mean average P build up was comparable to that reported by Withers et al. (2001) for arable (18.05 kg P ha^−1^ y^−1^) but was underestimated for the grassland (3.61 kg P ha^−1^ y^−1^) (Table 3). Other studies also found greater P surplus for grasslands in the UK ranging from 14 to 26 kg P ha^−1^ y^−1^ from farm to region (CAS, 1978; Brouwer et al. 1995; Smith et al., 1995). The difference in P balance between the simulated and that reported by Withers et al. (2001) is mainly due to high simulated P uptake by grass. However, simulated annual P uptake (30 kg ha^−1^) is similar to that reported elsewhere for grassland systems (Haygarth et al., 1998).

## 5 Conclusions

This paper describes an agricultural model (Roth-CNP) that was developed as part of an Integrated Model (LTLS-IM) to simulate the cycles of C, N and P for the whole of UK, comprising terrestrial, hydrological and hydro-chemical model over the long-term period from 1800 to the present. The Roth-CNP model summarises the CNP cycling in an agricultural ecosystem by aggregating soil and crop processes using a daily to monthly timestep. The model simulated crop and grass yields and estimated SOC stocks, DOC and POC losses, and nutrient fluxes (NH_4_-N, NO_3_-N and PO_4_-P) spatially across the whole UK taking into account the biophysical characteristics at each location. The simulated trends of crop yield are comparable to those reported by national agricultural statistics for the same period. Overall, arable land in the UK lost SOC between 1800 and the present day whereas under grassland, SOC stock increased over the same period. This is due to the fact that SOC builds up when a soil with a low initial SOC is planted to permanent grass and it may decrease under arable crops. Simulated N losses were comparable to losses/surpluses reported in the literature. Similarly, P dynamics including P loss and P surpluses were comparable to the literature reports although the P surplus was underestimated for the grass. The model results from the historical to current period show an increase in crop and grass yields especially due to increases in rates of mineral fertilizer application. This has resulted in large positive mineral N and P balances in the soil particularly during the post-war years and the period of the green revolution (1950–1980). Fertilizer inputs largely stabilised or decreased thereafter especially in improved grass and hereon there was increasing impact on nutrient losses rather than on yield. These results clearly show that the model can be used to explore the implications of different management options on crop or grass yields and the nutrient stocks and fluxes at the regional and national scales. The spatial variability of different variables (yield, SOC stock changes and nutrient fluxes) can be attributed to a combination of soil and weather factors. Although crop yields did not show any distinct spatial pattern between different periods of the simulation, in general, simulated crop yields were higher in the central and south west England where there was high rainfall and higher incidence of solar radiation during the growing season. Grass yields were higher in the western parts where rainfall is well distributed throughout the year. Soil organic carbon change did not follow any specific pattern with respect to SOC stock. But the N loss follows the spatial variation of rainfall with a higher loss in the western parts of the UK where rainfall is also higher c). However, no such pattern has been observed for P loss. In summary, the relatively simple agriculture model described in this paper was able to capture variability in the dynamics of CNP at the national scale once coupled to other large scale models of hydrology and soil erosion and driven by atmospheric deposition. The simulation results presented in this study can help farmers to see the effect of agriculture in their region within larger contexts of time and space to understand the consequence of their activities. For a policy maker, this study may help to design policies targeted at the regional level to curb negative impacts of agriculture on the sustainability of the environment. The model could be applied at subnational or catchment scale to attempt to optimise multiple stakeholder interests and for projecting forwards the plausible outcomes under different scenarios of climate and management.

The following are the supplementary data related to this article.

Supplementary information Image 1

Appendix I Description of different processes in Roth-CNP model. Appendix I

## Footnotes

1 SOM is often expressed as carbon content and is equal to SOC * 1.724

## References

[bb0005] ADAS (2007). Nitrogen Output of Livestock Excreta. ADAS Report to Defra – Supporting Paper F2 for the Consultation on Implementation of the Nitrates Directive in England.

[bb0010] Al-Adamat R., Rawajfih Z., Easter M., Paustian K., Coleman K., Milne E., Falloon P., Powlson D.S., Batjes N.H. (2007). Predicted soil organic carbon stocks and changes in Jordan between 2000 and 2030 made using the GEFSOC modelling system. Agric. Ecosyst. Environ..

[bb0015] Allen R.C. (1999). Tracking the agricultural revolution in England. Econ. Hist. Rev..

[bb0020] Allen R.C. (2008). The nitrogen hypothesis and the English agricultural revolution: a biological analysis. J. Econ. Hist..

[bb0025] Archer J. (1985). Crop Nutrition and Fertiliser Use.

[bb0030] Bell V.A., Kay A.L., Jones R.G., Moore R.J., Reynard N.S. (2009). Use of soil data in a grid-based hydrological model to estimate spatial variation in changing flood risk across the UK. J. Hydrol..

[bb0035] Bellamy P.H., Loveland P.J., Bradley R.I., Lark R.M., Kirk G.J.D. (2005). Carbon losses from all soils across England and Wales 1978-2003. Nature.

[bb0040] Bhogal A., Chambers B.J., Whitmore A.P., Young I. (2010). Organic manure and crop organic carbon returns – effects on soil quality: SOIL-QC. Final Report for Defra Project SP0530.

[bb0045] Bohlool B.B., Ladha J.K., Garrity D.P., George T. (1992). Biological nitrogen fixation for sustainable agriculture: a perspective. Plant Soil.

[bb0050] Bouwman L., Goldewijk K.K., Van Der Hoek K.W., Beusen A.H.W., Van Vuuren D.P., Willems J., Rufino M.C., Stehfest E. (2013). Exploring global changes in nitrogen and phosphorus cycles in agriculture induced by livestock production over the 1900{\textendash}2050 period. Proc. Natl. Acad. Sci..

[bb0055] Bowes M.J., Leach D.V., House W.A. (2005). Seasonal nutrient dynamics in a chalk stream: the river Frome, Dorset, UK. Sci. Total Environ..

[bb0060] Brilli L., Bechini L., Bindi M., Carozzi M., Cavalli D., Conant R., Dorich C.D., Doro L., Ehrhardt F., Farina R., Ferrise R., Fitton N., Francaviglia R., Grace P., Iocola I., Klumpp K., Léonard J., Martin R., Massad R.S., Recous S., Seddaiu G., Sharp J., Smith P., Smith W.N., Soussana J.-F., Bellocchi G. (2017). Review and analysis of strengths and weaknesses of agro-ecosystem models for simulating C and N fluxes. Sci. Total Environ..

[bb3520] Brouwer F., Godeschalk F.E., Hellegers P., Kelholt H.J. (1995). Mineral balances at farm Level in the European Union. Agricultural Economics Research Institute (LEI-DLO).

[bb1520] CAS (1978). Phosphorus: a Resource for UK Agriculture.

[bb0065] Coleman K., Jenkinson D.S., Crocker G.J., Grace P.R., Klír J., Körschens M. (1997). Simulating trends in soil organic carbon in long-term experiments using RothC-26.3. Geoderma.

[bb2040] Coleman K., Jenkinson D.S. (1999). RothC-26.3 - A Model for the turnover of carbon in soil: model description and windows users guide.

[bb0070] Coleman K., Muhammed S.E., Milne A.E., Todman L.C., Dailey A.G., Glendining M.J. (2017). The landscape model: a model for exploring trade-offs between agricultural production and the environment. Sci. Total Environ..

[bb0075] Cooke G.W. (1980). Changes in Fertilizer Use in the UK From 1950 to 1980.

[bb0080] Cui S., Shi Y., Groffman P.M., Schlesinger W.H., Zhu Y.-G. (2013). Centennial-scale analysis of the creation and fate of reactive nitrogen in China (1910–2010). Proc. Natl. Acad. Sci..

[bb0085] D'Arcy P., Carignan R. (1997). Influence of catchment topography on water chemistry in southeastern Québec shield lakes. Can. J. Fish. Aquat. Sci..

[bb0090] Davies J.A.C., Tipping E., Rowe E.C., Boyle J.F., Graf Pannatier E., Martinsen V. (2016). Long-term P weathering and recent N deposition control contemporary plant-soil C, N, and P. Glob. Biogeochem. Cycles.

[bb0095] DEFRA (2010). Fertilizer Manual (RB209).

[bb0100] DEFRA (2011). ARCHIVE: Nitrate Vulnerable Zones (NVZs) - Guidance for Farmers. Standard Values, Manure Sampling (Leaflet 3 - PB12736c). http://adlib.everysite.co.uk/adlib/defra/content.aspx?doc=251225&id=251492.

[bb0105] DEFRA (2011). The British survey of fertilizer practice. Fertilizer Use on Fram Crops for Crop Year 2010.

[bb0110] DEFRA (2014). https://www.gov.uk/government/uploads/system/uploads/attachment_data/file/338225/auk-chapter08-30jul14.xls.

[bb0115] deVries F.W.T.P. (1989). Simulation of Ecophysiological Processes of Growth in Several Annual Crops.

[bb0120] Dragosits U., Sutton M.A., Place C.J., Bayley A.A. (1998). Modelling the spatial distribution of agricultural ammonia emissions in the UK. Environ. Pollut..

[bb0125] Dungait J.A.J., Cardenas L.M., Blackwell M.S.A., Wu L., Withers P.J.A., Chadwick D.R. (2012). Advances in the understanding of nutrient dynamics and management in UK agriculture. Sci. Total Environ..

[bb0130] Edwards K.J., Hirons K.R. (1984). Cereal pollen grains in pre-elm decline deposits: implications for the earliest agriculture in Britain and Ireland. J. Archaeol. Sci..

[bb0135] Galloway J.N., Dentener F.J., Capone D.G., Boyer E.W., Howarth R.W., Seitzinger S.P. (2004). Nitrogen cycles: past, present, and future. Biogeochemistry.

[bb0140] Hatfield J.L., Boote K.J., Kimball B.A., Ziska L.H., Izaurralde R.C., Ort D., Thomson A.M., Wolfe D.W. (2011). Climate impacts on agriculture: implications for crop production. Agron. J..

[bb9100] Haygarth P.M., Chapman P.J., Jarvis S.C., Smith R.V. (1998). Phosphorus budgets for two contrasting farming systems in the UK. Soil Use Manag..

[bb0145] Haynes R.J., Williams P.H. (1993). Nutrient cycling and soil fertility in the grazed pasture ecosystem. Adv. Agron..

[bb0150] Hellsten S., Dragosits U., Place C.J., Vieno M., Dore A.J., Misselbrook T.H. (2008). Modelling the spatial distribution of ammonia emissions in the UK. Environ. Pollut..

[bb0155] Herridge D.F., Peoples M.B., Boddey R.M. (2008). Global inputs of biological nitrogen fixation in agricultural systems. Plant Soil.

[bb0160] Hofstra N., Bouwman A.F. (2005). Denitrification in agricultural soils: summarizing published data and estimating global annual rates. Nutr. Cycl. Agroecosyst..

[bb0165] Høgh-Jensen H., Schjoerring J.K. (1994). Measurement of biological dinitrogen fixation in grassland: comparison of the enriched 15N dilution and the natural 15N abundance methods at different nitrogen application rates and defoliation frequencies. Plant Soil.

[bb0170] Hood A.E.M. (1982). Fertilizer trends in relation to biological productivity within the UK. Philos. Trans. R. Soc. Lond. B.

[bb0175] Hooda P.S., Edwards A.C., Anderson H.A., Miller A. (2000). A review of water quality concerns in livestock farming areas. Sci. Total Environ..

[bb0180] Hopkins D.W., Waite I.S., McNicol J.W., Poulton P.R., Macdonald A.J., O'Donnell A.G. (2009). Soil organic carbon contents in long-term experimental grassland plots in the UK (palace leas and park grass) have not changed consistently in recent decades. Glob. Chang. Biol..

[bb0185] Hough M.N., Jones R.J.A. (1997). The United Kingdom meteorological office rainfall and evaporation calculation system: MORECS version 2.0-an overview. Hydrol. Earth Syst. Sci..

[bb0190] Hunt D.T.E., Dee A.S., Oakes D.B. (2004). Updating an estimate of the sources of nitrogen to UK waters – phase 2. Defra Final Report for Project WT03016.

[bb3040] Jenkinson D.S., Coleman K. (2008). The turnover of organic carbon in subsoils. Part 2. Modelling carbon turnover. Eur. J. Soil Sci.

[bb0200] Johnston A.E., Poulton P.R., Coleman K., Donald L.S. (2009). Chapter 1 soil organic matter: its importance in sustainable agriculture and carbon dioxide fluxes. Advances in Agronomy.

[bb0205] Jones C.A., Dyke P.T., Williams J.R., Kiniry J.R., Benson V.W., Griggs R.H. (1991). EPIC: an operational model for evaluation of agricultural sustainability. Agric. Syst..

[bb0210] Jones J.W., Hoogenboom G., Porter C.H., Boote K.J., Batchelor W.D., Hunt L.A., Wilkens P.W., Singh U., Gijsman A.J., Ritchie J.T. (2003). The DSSAT cropping system model. Eur. J. Agron..

[bb0215] Kamoni P.T., Gicheru P.T., Wokabi S.M., Easter M., Milne E., Coleman K., Falloon P., Paustian K. (2007). Predicted soil organic carbon stocks and changes in Kenya between 1990 and 2030. Agric. Ecosyst. Environ..

[bb0220] Lantinga EA, Keuning JA, Groenwold J, Deenen PJAG. Distribution of excreted nitrogen by grazing cattle and its effects on sward quality, herbage production and utilization. In: Van Der Meer HG, Unwin RJ, Van Dijk TA, Ennik GC, editors. Animal Manure on Grassland and Fodder Crops. Fertilizer or Waste? Proceedings of an International Symposium of the European Grassland Federation, Wageningen, The Netherlands, 31 August–3 September 1987. Springer Netherlands, Dordrecht, 1987, pp. 103–117.

[bb0225] Leenhardt D., Angevin F., Biarnès A., Colbach N., Leenhardt D., Angevin F., Biarnès A., Colbach N., De- C.M. (2010). Describing and locating cropping systems on a regional scale. A review. Agronomy for Sustainable Development.

[bb0230] Lesschen J.P., Stoorvogel J.J., Smaling E.M.A., Heuvelink G.B.M., Veldkamp A. (2007). A spatially explicit methodology to quantify soil nutrient balances and their uncertainties at the national level. Nutr. Cycl. Agroecosyst..

[bb0235] Li C., Frolking S., Frolking T.A. (1992). A model of nitrous oxide evolution from soil driven by rainfall events: 1. Model structure and sensitivity. J. Geophys. Res. Atmos..

[bb0240] Liu J., You L., Amini M., Obersteiner M., Herrero M., Zehnder A.J.B., Yang H. (2010). A high-resolution assessment on global nitrogen flows in cropland. Proc. Natl. Acad. Sci..

[bb0245] Liu Y., Wu L., Watson C.A., Baddeley J.A., Pan X., Zhang L. (2013). Modeling biological dinitrogen fixation of field pea with a process-based simulation model. Agron. J..

[bb0250] Lobell D.B., Schlenker W., Costa-Roberts J. (2011). Climate trends and global crop production since 1980. Science.

[bb0255] Long S.P., Ainsworth E.A., Leakey A.D.B., Morgan P.B. (2005). Global food insecurity. Treatment of major food crops with elevated carbon dioxide or ozone under large-scale fully open-air conditions suggests recent models may have overestimated future yields. Philos. Trans. R. Soc. Lond. Ser. B Biol. Sci..

[bb0260] Long S.P., Ainsworth E.A., Leakey A.D.B., Nösberger J., Ort D.R. (2006). Food for thought: lower-than-expected crop yield stimulation with rising CO2 concentrations. Science.

[bb0265] Lord E.I., Anthony S.G., Goodlass G. (2002). Agricultural nitrogen balance and water quality in the UK. Soil Use Manag..

[bb0270] Lüscher A., Mueller-Harvey I., Soussana J.F., Rees R.M., Peyraud J.L. (2014). Potential of legume-based grassland–livestock systems in Europe: a review. Grass Forage Sci..

[bb0275] MacDonald G.K., Bennett E.M., Potter P.A., Ramankutty N. (2011). Agronomic phosphorus imbalances across the worlds croplands. Proc. Natl. Acad. Sci..

[bb0280] MAFF (1998). SUNDIAL-FRS User Guide Version 1.0.

[bb0285] Manston R., Vagg M.J. (2009). Urinary phosphate excretion in the dairy cow. J. Agric. Sci..

[bb0290] Manzoni S., Porporato A. (2009). Soil carbon and nitrogen mineralization: theory and models across scales. Soil Biol. Biochem..

[bb0295] Marks H.F., Britton D.K. (1989). A Hundred Years of British Food and Farming: A Statistical Survey.

[bb0300] McCown R.L., Hammer G.L., Hargreaves J.N.G., Holzworth D.P., Freebairn D.M. (1996). APSIM: a novel software system for model development, model testing and simulation in agricultural systems research. Agric. Syst..

[bb0305] McGechan M.B., Topp C.F.E. (2004). Modelling environmental impacts of deposition of excreted nitrogen by grazing dairy cows. Agric. Ecosyst. Environ..

[bb0310] McGill W.B., Powlson D.S., Smith P., Smith J.U. (1996). Review and Classification of ten Soil Organic Matter (SOM) Models. Evaluation of Soil Organic Matter Models.

[bb0315] Monteith J.L., Rabbinge R., Goudriaan J., Van Keulen H., Penning de Vries F.W.T., Van Laar H.H. (1990). Conservative behaviour in the response of crops to water and light. Theoretical Production Ecology: Reflections and Prospects.

[bb0320] Monteith J.L., Moss C.J. (1977). Climate and the efficiency of crop production in Britain [and discussion]. Philos. Trans. R. Soc. Lond. Ser. B Biol. Sci..

[bb0325] Morris J., Audsley E., Wright I.A., McLeod J., Pearn K., Angus A. (2005). Agricultural Futures and Implications for the Environment. Defra Research Project IS0209. http://hppt/www.silsoe.cranfield.ac.uk.

[bb0330] Musel A. (2009). Human appropriation of net primary production in the United Kingdom, 1800–2000: changes in society's impact on ecological energy flows during the agrarian–industrial transition. Ecol. Econ..

[bb0335] Naden P., Bell V., Carnell E., Tomlinson S., Dragosits U., Chaplow J. (2016). Nutrient fluxes from domestic wastewater: a national-scale historical perspective for the UK 1800–2010. Sci. Total Environ..

[bb0340] Nicholson F.A., Chambers B.J., Smith K.A. (1996). Nutrient composition of poultry manures in England and Wales. Bioresour. Technol..

[bb0345] Nievinski F.G. (2009). Ray tracing options to mitigate the neutral atmosphere delay in GPS. Technical Report.

[bb0350] Nix (2003). Farm Management Pocketbook. 34th Edition.

[bb0355] Nolan B.T., Stoner J.D. (2000). Nutrients in Groundwaters of the conterminous United States, 1992−1995. Environ. Sci. Technol..

[bb0360] Ogle S.M., Jay Breidt F., Eve M.D., Paustian K. (2003). Uncertainty in estimating land use and management impacts on soil organic carbon storage for US agricultural lands between 1982 and 1997. Glob. Chang. Biol..

[bb0365] Orr R.J., Tallowin J.R.B., Griffith B.A., Rutter S.M. (2014). Effects of livestock breed and rearing experience on foraging behaviour of yearling beef cattle grazing unimproved grasslands. Grass Forage Sci..

[bb0370] Overton M. (1996). Agricultural Revolution in England: The Transformation of the Agrarian Economy 1500–1850.

[bb0375] Parton W.J., Hartman M., Ojima D., Schimel D. (1998). DAYCENT and its land surface submodel: description and testing. Glob. Planet. Chang..

[bb0380] Pathak H., Mohanty S., Jain N., Bhatia A. (2010). Nitrogen, phosphorus, and potassium budgets in Indian agriculture. Nutr. Cycl. Agroecosyst..

[bb0385] Penman H.L. (1948). Natural evaporation from open water, bare soil and grass. Proc. R. Soc. London, Ser. A.

[bb0390] Powlson D.S., Jenkinson D.S., Harrison A.F., Ineson P., Heal O.W. (1990). Quantifying inputs of non-fertilzer nitrogen into an agro-ecosystem. Nutrient Cycling in Terrestrail Ecosystems: Field Methods, Application and Interpretation.

[bb1055] Powlson D.S., Gregory P.J., Whalley W.R., Quinton J.N., Hopkins D.W., Whitmore A.P., Hirsch P.R., Goulding K.W.T. (2011). Soil management in relation to sustainable agriculture and ecosystem services. Food Policy.

[bb0395] Pretty J.N., Brett C., Gee D., Hine R.E., Mason C.F., Morison J.I.L. (2000). An assessment of the total external costs of UK agriculture. Agric. Syst..

[bb0400] Reynolds B., Chamberlain P.M., Poskitt J., Woods C., Scott W.A., Rowe E.C. (2013). Countryside survey: national “soil change” 1978–2007 for Topsoils in great Britain—acidity, carbon, and Total nitrogen status. Vadose Zone J..

[bb0405] Rosen M.R., Reeves R.R., Green S., Clothier B., Ironside N. (2004). Prediction of groundwater nitrate contamination after closure of an unlined sheep feedlot. Vadose Zone J..

[bb0410] Rozemeijer J.C., Broers H.P., FCv Geer, Bierkens M.F.P. (2009). Weather-induced temporal variations in nitrate concentrations in shallow groundwater. J. Hydrol..

[bb0415] Sakadevan K., Mackay A.D., Hedley M.J. (1993). Influence of sheep excreta on pasture uptake and leaching losses of Sulphur, nitrogen and potassium from grazed pastures. Aust. J. Soil Res..

[bb0420] Sanderson M.A., Brink G., Stout R., Ruth L. (2013). Grass–legume proportions in forage seed mixtures and effects on herbage yield and Weed abundance. Agron. J..

[bb0425] Shand C.A., Williams B.L., Dawson L.A., Smith S., Young M.E. (2002). Sheep urine affects soil solution nutrient composition and roots: differences between field and sward box soils and the effects of synthetic and natural sheep urine. Soil Biol. Biochem..

[bb0430] Shibu M.E., Leffelaar P.A., van Keulen H., Aggarwal P.K. (2010). LINTUL3, a simulation model for nitrogen-limited situations: application to rice. Eur. J. Agron..

[bb0435] Smil V. (1999). Nitrogen in crop production: an account of global flows. Glob. Biogeochem. Cycles.

[bb5520] Smith R.V., Lennox S.D., Jordan C., Foy R.H., McHale E. (1995). Increase in soluble phosphorus transported in drainflow from a grassland catchment in response to soil phosphorus accumulation. Soil Use Manage.

[bb9000] Smith P., Falloon P., Coleman K., Smith J., Piccolo M.C., Cerri C., Bernoux M., Jenkinson D., Ingram J., Szabo J., Pasztor L., Lal R., Kimble J.M., Stewart B.A. (2000). Modeling soil carbon dynamics in tropical ecosystems. Global Climate Change and Tropical Ecosystems.

[bb0440] Smith J., Gottschalk P., Bellarby J., Chapman S., Lilly A., Towers W., Bell J., Coleman K., Nayak D., Richards M., Hillier J., Flynn H., Wattenbach M., Aitkenhead M., Yeluripati J., Farmer J., Milne R., Thomson A., Evans C., Whitmore A., Falloon P., Smith P. (2010). Estimating changes in Scottish soil carbon stocks using ECOSSE. II. Application. Clim. Res..

[bb0445] Stöckle C.O., Donatelli M., Nelson R. (2003). CropSyst, a cropping systems simulation model. Eur. J. Agron..

[bb0450] Stuart M.E., Gooddy D.C., Bloomfield J.P., Williams A.T. (2011). A review of the impact of climate change on future nitrate concentrations in groundwater of the UK. Sci. Total Environ..

[bb0455] Sylvester-Bradley R., Wiseman J. (2005). Yields of Farmed Species: Constraints and Opportunities in the 21st Century. Proceedings of a University of Nottingham Easter School Series, June 2004, Sutton Bonington, UK. Yields of Farmed Species: Constraints and Opportunities in the 21st Century. Proceedings of a University of Nottingham Easter School Series. June.

[bb0460] Tian H., Lu C., Yang J., Banger K., Huntzinger D.N., Schwalm C.R., Michalak A.M., Cook R., Ciais P., Hayes D., Huang M., Ito A., Jain A.K., Lei H., Mao J., Pan S., Post W.M., Peng S., Poulter B., Ren W., Ricciuto D., Schaefer K., Shi X., Tao B., Wang W., Wei Y., Yang Q., Zhang B., Zeng N. (2015). Global patterns and controls of soil organic carbon dynamics as simulated by multiple terrestrial biosphere models: current status and future directions. Glob. Biogeochem. Cycles.

[bb0465] Tipping E., Rowe E.C., Evans C.D., Mills R.T.E., Emmett B.A., Chaplow J.S. (2012). N14C: a plant–soil nitrogen and carbon cycling model to simulate terrestrial ecosystem responses to atmospheric nitrogen deposition. Ecol. Model..

[bb0470] Tipping E., Benham S., Boyle J.F., Crow P., Davies J., Fischer U., Guyatt H., Helliwell R., Jackson-Blake L., Lawlor A.J., Monteith D.T., Rowe E.C., Toberman H. (2014). Atmospheric deposition of phosphorus to land and freshwater. Environ. Sci. Process. Impacts.

[bb0475] Tipping E., Davies J.A.C., Henrys P.A., Kirk G.J.D., Lilly A., Dragosits U., Carnell E.J., Dore A.J., Sutton M.A., Tomlinson S.J. (2017). Long-term increases in soil carbon due to ecosystem fertilization by atmospheric nitrogen deposition demonstrated by regional-scale modelling and observations. Sci. Rep..

[bb0480] Wang Y.P., Law R.M., Pak B. (2010). A global model of carbon, nitrogen and phosphorus cycles for the terrestrial biosphere. Biogeosciences.

[bb0485] Wang G., Zhang W., Sun W., Li T., Han P. (2017). Modeling soil organic carbon dynamics and its driving factors in global main cereal cropping systems. Atmos. Chem. Phys. Discuss..

[bb0490] Wheeler J.L. (1959). The effect of sheep urine on the GERIMINATION and early establishment of a common WEED grass. Grass Forage Sci..

[bb0495] Whetham E.H. (1970). THE MECHANISATION OF BRITISH FARMING, 1910–1945. J. Agric. Econ..

[bb0500] White P.J., Hammond J.P. (2007). Updating the estimate of the sources of phosphorus in UK waters. Defra Final Report for Project WT0701CSF.

[bb0505] Whitehead D.C. (1995). Grassland Nitrogen.

[bb0510] Whitmore A.P., Bradbury N.J., Johnson P.A. (1992). Potential contribution of ploughed grassland to nitrate leaching. Agric. Ecosyst. Environ..

[bb0515] Williams P.H., Haynes R.J. (1995). Effect of sheep, deer and cattle dung on herbage production and soil nutrient content. Grass Forage Sci..

[bb0520] Withers P.J.A., Edwards A.C., Foy R.H. (2001). Phosphorus cycling in UK agriculture and implications for phosphorus loss from soil. Soil Use Manag..

[bb0525] Wolf J. (2012). User Guide for LINTUL4 and LINTUL4V: Simple Generic Model for Simulation of Crop Growth Under Potential, Water Limited and Nitrogen Limited Conditions Wageningen UR, Wageningen.

[bb0530] Woodbridge J., Fyfe R.M., Roberts N., Downey S., Edinborough K., Shennan S. (2014). The impact of the Neolithic agricultural transition in Britain: a comparison of pollen-based land-cover and archaeological 14C date-inferred population change. J. Archaeol. Sci..

[bb0535] Zaehle S. (2013). Terrestrial nitrogen-carbon cycle interactions at the global scale. Philos. Trans. R. Soc. Lond. Ser. B Biol. Sci..

