# Supplementary material for: Impact of two centuries of intensive agriculture on soil carbon, nitrogen and phosphorus cycling in the UK
Source: Sci Total Environ. 2018 Sep 1;634:1486–504. doi: 10.1016/j.scitotenv.2018.03.378 (PMC5981008; doi:10.1016/j.scitotenv.2018.03.378)
Supplement: Supplementary file 1 — Supplementary information [file mmc1.docx]

**Supplementary Material**

**Impact of two centuries of intensive agriculture on soil carbon, nitrogen and phosphorus cycling in the UK**

**Shibu E. Muhammed^1^*, Kevin Coleman^1^, Lianhai Wu^2^, Victoria A. Bell^3^, Jessica A. C. Davies^4^,** **John, N Quinton^4^, Edward J. Carnell^5^, Samuel J. Tomlinson^5^, Anthony J. Dore^5^, Ulrike Dragosits^5^, Pamela S. Naden^3^, Margaret J. Glendining^1^, Edward Tipping^6^, Andrew P. Whitmore^1^**

^1^ Rothamsted Research, Harpenden, Hertfordshire AL5 2JQ, UK

^2^ Rothamsted Research, North Wyke, EX20 2SB, UK

^3^ Centre for Ecology & Hydrology, Wallingford, Oxfordshire, OX10 8BB, UK

^4^ Lancaster Environment Centre, Lancaster University, LA1 4YQ, UK

^5^ Centre for Ecology & Hydrology, Bush Estate, Penicuik, EH26 0QB, UK

^6^ Centre for Ecology & Hydrology, Library Avenue, Lancaster LA1 4AP, UK

*Keywords: Roth-CNP, Integrated model, crops, nutrient flux, leaching*

*Corresponding author

E-mail address [shibu.muhammed@rezatec.com](mailto:shibu.muhammed@rezatec.com)

# S1. Atmospheric deposition model

Atmospheric deposition of N was calculated with FRAME, a Lagrangian atmospheric chemistry transport model which is driven by annually averaged meteorology. The precipitation data used to calculate wet deposition used a 20 year average annual precipitation map calculated from the UK Met Office precipitation monitoring network and an average wind direction frequency rose which was calculated for a 10-year period from radiosonde data ([Dore et al., 2006](#_ENREF_10)). The model has 33 vertical layers with variable depth from 1 m at the surface to 100 m for the upper layer and a horizontal resolution of 5 km x 5 km which covers the British Isles. Emissions of SO_2_, NO_x_ and NH_3_ were input to the model and a chemical transformation scheme was employed to calculate the oxidation of primary gases and their reaction to form particulates (ammonium sulphate and ammonium nitrate). The model calculated dry deposition to six different land categories (forest, semi-natural grassland, improved grassland, arable, urban and water) using a big leaf model employing a resistance analogy to estimate dry deposition velocities ([Smith et al., 2000](#_ENREF_22)). A European scale version of the model with a horizontal resolution of 50 km x 50 km was used to generate the boundary conditions for air concentrations used to initialize the national scale simulation. The model performance is evaluated on an annual basis to compare modelled concentrations of gases, particulates and precipitation concentrations with measurements from the UK national monitoring networks ([Dore et al., 2015](#_ENREF_9)) and has been demonstrated to be ‘fit for purpose’ as a deposition model. The model has been used to generate source-receptor matrices for use in integrated assessment modelling to provide advice to policy makers on the most cost-effective pollutant emissions abatement strategy for protection of both human health and natural ecosystems.

# S2. Land cover data

Modern day land cover was determined from the Land Cover Map 2007 (([Morton et al.](#_ENREF_18)), 2011), which reports arable and improved grassland land use types. This was processed to produce land use fractions for each 5 x 5 km grid cell. This was combined with the Dudley Stamp Land Utilisation Survey of Britain ([Stamp, 1941](#_ENREF_23)), which was completed in 1931 and reports land use including pasture and arable at a 1:625,000 scale. A historical land cover data series (1800-2007) was prepared by combining these maps with knowledge-based rules (Figure S2.2). For example, the total arable area across the UK is known to have expanded between 1931 and 2007. This expansion mainly occurred within a very short period during and after the Second World War ([Marks and Britton, 1989](#_ENREF_15)). If the fraction of agricultural land had expanded between the 1931 and 2007 surveys for any grid cell, a general assumption was made that this expansion would have taken place by 1950, and this date was used in the model. The arable expansion in each cell was assumed to have been a conversion of improved grassland to arable first, and then if insufficient pasture land was available, rough grassland. If an area was designated as arable or improved pasture in 1931, it was assumed that it had been so since 1800 – the start date of the simulations. These broad land cover types (Figure S2.1) were then differentiated into different landuse types based on crop types and livestock densities.

# S3. Livestock and landuse data

Livestock populations and agricultural land use data were estimated for four time slices 1900, 1950, 1970 and 1990. Estimates were based on agricultural census data and were distributed using the AENEID model (Dragosits et al. 2006; Hellsten et al. 2008) with the land cover data summarised in S2 as input.

Agricultural statistics for the years 1900 and 1950 were based on county level agricultural census data downloaded for Great Britain from ([Britain, 2014](#_ENREF_6)) and for Northern Ireland from ([Clarkson et al., 1997](#_ENREF_7)). The 1901 census was used as a proxy for the 1900 time slice. The 1950 estimates were based on the 1951 census for England and Wales, the 1961 census for Scotland and the 1952 census for Northern Ireland. The 1951/1961 county level data for Great Britain were scaled to official 1950 figures given in (FAO, 1955).

The 1970 and 1990 estimates for Great Britain were based on agricultural census data downloaded from Edina (2012) at a 2 km grid resolution (for 1971 and 1991, respectively). For Northern Ireland, county level data from the 1971 agricultural census were taken from ([Dowling et al., 1998](#_ENREF_11)) for 1970 and the 1991 census was taken at the Rural District level for 1990.

# S4. Hydrology model

The hydrological model ([Bell et al., in prep](#_ENREF_3)) is based on a 5km implementation of the hydrological modelling framework (HMF) ([Crooks et al., 2014](#_ENREF_8)), with parallel surface and sub-surface kinematic wave lateral flow routing scheme similar to the earlier G2G ([Bell *et al.*, 2007](#_ENREF_2)) and allows for a variety of runoff-production schemes. The runoff-production scheme used here is similar to that implemented in the G2G ([Bell et al., 2009](#_ENREF_2)), which maintains water storage in a soil column by balancing inputs from rainfall with “losses” from evaporation, soil drainage, and runoff from surface and sub-surface. Hydraulic properties (saturation and residual water contents) provide estimates of maximum and residual water storage of each 5×5km soil column. The hydraulic parameters are derived using a Van Genuchten scheme ([Van Genuchten, 1980](#_ENREF_24)) applied to Harmonised World Soil Database (HWSD, <http://www.fao.org/docrep/018/aq361e/aq361e.pdf>) spatial datasets of soil texture, available at a 30 arc-second (~1 km) spatial resolution and re-gridded to 5km. In order to ensure that each grid-square generates realistic quantities of saturation-excess surface runoff even when it is not fully saturated, the probability-distributed soil moisture store formulation of Moore ([1985](#_ENREF_16); [Moore, 2007](#_ENREF_17)) has been invoked within each grid-square following ([Bell et al., 2009](#_ENREF_2)). For hydrological modelling of water quantity, any effect of land-cover on runoff production and downstream river flows is neglected, except in urban/suburban area where available soil storage is reduced leading to a faster response to rainfall.

# S5. Erosion

Erosion from agricultural land uses is estimated here using a semi-empirical approach. The loss of soil from the land to the waterway via water erosion *S* (kg m^2^ s‾^1^) is assumed to be related to: soil protection, *P*, (principally vegetation cover), soil erodibility, *K*, the erosive energy of flowing water, *E*, and connectivity to stream, Y, as follows:

*S* *= E ×K ×(1-P) × Y*

Erosive energy, *E (*J m^2^ s‾^1^*)*, is based on a standard calculation of stream power (Bagnold, 1966), that uses an estimate of overland flow provided by the hydrology model (see S4), and topography, here provided on the 5 x 5km grid by the HWSD which gives frequencies for 8 categories of slope.

Soil erodibility, *K* (kg J⁻^1^) determines the rate of soil detachment per unit of erosive energy applied from water flow and is assumed to be largely related to soil texture. In this instance, the values of K are based upon the guide values compiled for EUROSEM (Morgan et al. 1998), and soil texture is derived from the HWSD.

The vegetation protection term (0≤P≤1, where 1 represents full protection) is used to scale the erosion. In arable areas *P* is estimated from crop LAI defined on a monthly basis, where it is assumed that an LAI of 5 or more equates to full protection. In improved grasslands, grazing density data (ρ) (see S3) is used to estimate the reduction of vegetation cover arising from sheep grazing. Connectivity to stream, Y, takes on a value between 0 and 1, and is based on drainage density, which is calculated from a DEM.

This erosion model is used to provide a broad estimate of the volume of soil, and associated nutrients, lost from land to waterways at a large scale and over a long timescale. It has relatively few parameters, which have been set using literature data. It neglects the effects of tillage, harvesting, wind erosion, subsoil loss, in-cell redistribution of soil, compaction or poaching, field tracks and paths, agricultural drainage, and buffer strips or hedgerows.

# S6. Roth-CNP model calibration and testing

We calibrated and tested the arable and grass land models using the data from long term experiments (LTE) from Broadbalk and Park Grass available since the 1840’s at Rothamsted (51° 48’ 36’’ N; 0° 22’ 30’’ W) (Table S 5.1). More details of the Broadbalk and Park Grass LTEs are given below:

*Broadbalk LTE*:

The Broadbalk LTE was started in 1843 on a field which had been in arable cropping for many years before ([Rothamsted-Research, 2006](#_ENREF_19)). The whole field was divided into 19 plots with different fertilizer and organic manure treatments. Until 1925, continuous winter wheat was grown every year on the whole experiment, except for occasional fallowing to control weeds. In 1926, the plots were divided into five sections (I-V) and fallows were introduced after every four years of wheat to combat weed problems. From 1968 onwards, these five sections were split to create a total of 10 sections (0-9) and rotations were introduced in some of these sections (2,3,4,5 and 7) by including break crops such as potato, oats and maize or fallow after one to three consecutive wheat crops. Winter wheat yield data from 1852-2010 was used in this paper. 1852 was chosen as the starting date, as the fertilizer treatments varied somewhat during the first few years of the experiment but in 1852 a permanent scheme was established which has continued, with minor modifications, to today. Data from 1852-1925 was for wheat grown continuously; from 1926-2010, yield data was from the first wheat in the rotational sections (section 3 and 5) only. In years when a first wheat was grown on both sections, the average yield was used. Yield values for potato and fodder maize were also available for the years in rotation (1968-1996 for potatoes, 1997-2010 for forage maize). We tested the model with selected fertilizer and manure treatments (Table S6.1). Initial SOC (1.0%, 23 cm depth) value in 1843 was estimated based on the measurements in 1865 when soils were first sampled ([*http://www.era.rothamsted.ac.uk/Broadbalk/bbk_open_access_soc*](http://www.era.rothamsted.ac.uk/Broadbalk/bbk_open_access_soc)). Soil samples were taken every now and then since then with more regular (5 to 6-year interval) sampling in the later years (1987 onwards). Nitrate leaching was measured after 157-years since the start of the experiment for over eight drainage seasons (1990 – 1998) ([Goulding et al., 2000](#_ENREF_13)). Soil is clay loam to silty clay loam in texture with a clay and silt content of 25 and 50%, respectively at surface 0-23 cm depth. Soils have been limed to maintain a pH of 7-7.5 since the 1950s. See [http://www.era.rothamsted.ac.uk/Broadbalk](http://www.era.rothamsted.ac.uk/Park) for more details.

*Park Grass LTE*:

The Park Grass experiment was established in 1856, on a field that had been under permanent grass for at least 100 years before ([Bowley et al., 2017](#_ENREF_5); [Rothamsted-Research, 2006](#_ENREF_19)). The experiment is a permanent grassland, it is never cultivated or sown. The model was tested with selected fertilizer and organic manure treatments (Table S6.1). Initial SOC (3.2%, 0-23cm depth) value in 1856 was estimated based on the SOC measurements in 1876. Soil samples have been taken periodically since then. The soil is silty clay loam in texture (23% clay, 52% silt) in the top 23 cm. Grass is cut in mid-June (first cut) and since 1875 it has been cut a second time in September-November. The model was tested against total yield (cut 1 plus cut 2) for 1856-2009, except for plot 14/2, which began in 1858. In 1960, there was a change in the harvest method for cut 1 and a correction factor was applied, so that yields are directly comparable with the earlier harvest method. See [Bowley et al. (2017](#_ENREF_5)) for details. Initial soil pH was 5.5 and this was regularly monitored and limed as required on subplots since 1903. We used the data from subplots which were limed to pH 6 under each treatment. Model results were compared with measured grass cut yields, soil organic carbon (SOC) and soil total nitrogen, (STN). See <http://www.era.rothamsted.ac.uk/Park> for more details.

*Model Inputs:*

Weather data (rainfall since 1853 and temperature from 1878 onwards) were available from the Electronic Rothamsted Archive (e-RA http://www.era.rothamsted.ac.uk). For missing values of temperature and rainfall prior to 1902, we used the 5 x 5 km grid cell encompassing Rothamsted (see section 2.2.2). Similarly, for atmospheric N deposition and hydrological information (soil moisture, AET, drainage and runoff) outputs from the hydrology model for the grid covering Rothamsted was used.

*Model Calibration*:

For calibration of the wheat crop module, we selected one of the Broadbalk plots (Plot 16) which received 96 kg N ha^−1^ up to 1984 increased to 288 kg N ha^−1^ from thereafter, with non-limiting P, K, Na and Mg. The crop model needed calibration particularly for older varieties. Only a few changes were made for the older varieties compared to the modern short-strawed varieties. These changes are reduced N sensitivity to applied N fertilizer and more biomass partitioning to roots and stems. To reduce the N sensitivity, the maximum N uptake rate for the older varieties was reduced from the current 150 kg N ha^−1^ month^−1^ (based on a daily rate of 5kg N ha^−1^ ([Semenov et al., 2007](#_ENREF_20)) to 30 kg ha^−1^ month^−1^ by calibration. The biomass partitioning for older varieties was also modified in relation to the new varieties by calibration by modifying biomass allocation to the stem and storage organs (Figure S6.1). [Waines and Ehdaie (2007](#_ENREF_25)) reported that pre-green revolution wheat varieties had a higher root biomass by about 1/3^rd^ and the values were adjusted accordingly.

Model calibration for old crop varieties show that grain, straw, aboveground biomass (AGB) and harvest index (HI) were closely following the measurements except for periods of 1860-1920 where the measured yields were substantially lower than periods before and after (Figure S6.1). This anomaly may arise due to the incidence of soil borne diseases such as take all (*Gaeumannomyces graminis*) and heavy weed infestation as a result of continuous wheat cultivation ([Austin et al., 1993](#_ENREF_1); [Dyke et al., 1982](#_ENREF_12)). Hence, from 1925 onwards, sequential fallows were introduced in Broadbalk to reduce the weed build up. However, the model did take into account any effect of pest and diseases on crop yields. Calibration results show an overall RMSE of 1.9 Mg ha‾^1^, 1.0 Mg C ha‾^1^ and 0.4 Mg C ha‾^1^  for wheat yield, SOC and STN (Table S6.2).

## S 6.1. Results of testing of arable system model (using measured data from the Broadbalk LTE)

Model results were compared with measured crop yields, SOC and STN graphically and statistically.

Simulated wheat yields closely matched the observed results in most of the plots we tested (Figure S6.2). Statistical comparison of the model and observed results show highest correlation for wheat yield in plot 15 (*r* =0.87) with an RMSE of 2.0 Mg ha‾^1^  (Table S6. 3). When all observed and simulated yields were compared (*r* =0.80, *M* =−1.0 and RMSE = 1.7 Mg ha‾^1^ ) results showed a good agreement to the observed although yields were slightly overestimated in the earlier part of the experiment (see Model Calibration for possible reasons). However, for the other crops: potato and fodder maize, simulated yield trends (r = −0.6 to 0.8 for potato; r = −0.98 to −0.5 for fodder maize) were less comparable to the measured (Table S6.3). However, when all the potato plots were combined, the yield trends were comparable (r = 0.8) with an overall RMSE of 1.9 Mg ha‾^1^ . Similarly, results were found to be better for fodder maize when the yield from all plots were pooled (r = 0.63; RMSE= 4.0 Mg ha‾^1^ ). Simulated SOC closely matched the trend of measured SOC in all the fertilizer treatments except plot 3, where the model slightly overestimated (Figure S6.3). The correlation between the simulated and observed SOC were highest for plot 22 (*r* =0.93) with an RMSE of 4.5 Mg C ha‾^1^  , where FYM were applied annually (Table S6.3). The overall mean difference between the observed and simulated showed an overestimation of SOC by the model compared to the observed (M= −1.7). Similar to SOC, overall simulated STN also showed a good fit to the observed with a mean difference (*M* =0.11) showing an underestimation of the model results. The model was also tested for NO_3_-N leaching available for a few years (1991-1998) from Broadbalk ([Goulding et al., 2000](#_ENREF_13)). Simulated annual NO3-N leaching was higher than measured in all the plots except that of plot 22 (Figure S6.4). Nitrate N loss by leaching measured during 1991-1998 was slightly overestimated in all the mineral fertilized treatments.

## S 6.2. Results of testing of grass land system model (using Park Grass LTE)

Measured and simulated annual grass yields varied between years in all the plots (Figure S6.5). Statistical evaluation of the grass yields showed a better correlation for plot 14/2 (*r* = 0.47) with a mean difference (*M*) of -1.7 and RMSE of 2.1 Mg ha‾^1^  than the other treatments (Table S6.4). Overall grass yield was slightly overestimated (*M* =−0.7) when all the plots were pooled (RMSE = 1.3 Mg ha‾^1^ ). Simulated SOC followed the observed trend for plot 13/2 where FYM was applied whereas it was underestimated for plots 3 and 14/2 (Figure S6.6). The trend of simulated STN was very similar to that of SOC whereas measured STN did not show much difference between the three treatments. In fact, measured STN in plot 3 (nil) was higher than that of plot 13/2 and 14/2, which were fertilized. It could be very likely that total carbon inputs from dead roots, etc. would be very similar for all the treatments in the measured, perhaps slightly less where fertilizer or FYM is applied, as less root is required to take up the nutrients. However, in the model, the root contribution to the soil may reduce in proportion to the total biomass.

# References

Austin R, Ford M, Morgan C, Yeoman D. Old and modern wheat cultivars compared on the Broadbalk wheat experiment. European Journal of Agronomy 1993; 2: 141-147.

Bell VA, Kay AL, Jones RG, Moore RJ, Reynard NS. Use of soil data in a grid-based hydrological model to estimate spatial variation in changing flood risk across the UK. Journal of Hydrology 2009; 377: 335-350.

Bell VA, Naden PS, Tipping E, Davies HN, Carnell E, Davies J, et al. Long-Term and Large-Scale simulations of C, N and P in UK freshwater systems. in prep.

Boons - Prins ER, de Koning GHJ, van Diepen CA. Crop-specific simulation parameters for yield forecasting across the European Community. Wageningen: CABO-DLO [etc.], 1993.

Bowley HE, Mathers AW, Young SD, Macdonald AJ, Ander EL, Watts MJ, et al. Historical trends in iodine and selenium in soil and herbage at the Park Grass Experiment, Rothamsted Research, UK. Soil Use and Management 2017; 33: 252-262.

Bagnold, R.A., 1966. An approach to the sediment transport problem from general physics. US government printing office.

Britain Vo. Historic agricultural data [WWW Document]. URL <http://www.visionofbritain.org.uk/data/dds_entity_page.jsp?ent=T_LAND> (accessed 9.11.14). 2014.

Clarkson LA, Kennedy L, Crawford EM, Dowling MW. Database of Irish Historical Statistics : Population, 1821-1911. [data collection]. UK Data Service. SN: 3578., 1997.

Crooks S, Kay A, Davies H, Bell V. From Catchment to National Scale Rainfall-Runoff Modelling: Demonstration of a Hydrological Modelling Framework. Hydrology 2014; 1: 63.

Dore AJ, Carslaw DC, Braban C, Cain M, Chemel C, Conolly C, et al. Evaluation of the performance of different atmospheric chemical transport models and inter-comparison of nitrogen and sulphur deposition estimates for the UK. Atmospheric Environment 2015; 119: 131-143.

Dore AJ, Vieno M, Fournier N, Weston KJ, Sutton MA. Development of a new wind-rose for the British Isles using radiosonde data, and application to an atmospheric transport model. Quarterly Journal of the Royal Meteorological Society 2006; 132: 2769-2784.

Dowling MW, Clarkson LA, Kennedy L, Crawford EM. Database of Irish Historical Statistics : Agricultural Statistics, 1911-1973. 1998.

Dyke G, George B, Johnston A, Poulton P, Todd A. The Broadbalk wheat experiment 1968-78: yields and plant nutrients in crops grown continuously and in rotation. Rothamsted Experimental Station Report for 1982; 2: 5-44.

Goulding KWT, Poulton PR, Webster CP, Howe MT. Nitrate leaching from the Broadbalk Wheat Experiment, Rothamsted, UK, as influenced by fertilizer and manure inputs and the weather. Soil Use and Management 2000; 16: 244-250.

Loague K, Green RE. Statistical and graphical methods for evaluating solute transport models: Overview and application. Journal of Contaminant Hydrology 1991; 7: 51-73.

Marks HF, Britton DK. A Hundred Years of British Food and Farming: A Statistical Survey: Taylor & Francis, 1989.

Moore RJ. The probability-distributed principle and runoff production at point and basin scales. Hydrological Sciences Journal 1985; 30: 273-297.

Moore RJ. The PDM rainfall-runoff model. Hydrology and Earth System Sciences Discussions 2007; 11: 483-499.

Morgan, R.P.C., Quinton, J.N., Smith, R.E., Govers, G., Poesen, J.W.A., Chisci, G. and Torri, D., 1998. The EUROSEM model. In *Modelling Soil Erosion by Water* (pp. 389-398). Springer, Berlin, Heidelberg.

Morton D, Rowland C, Wood C, Meek L, Marston C, Smith G, et al. Final Report for LCM2007 – the New UK Land Cover Map. CS Technical Report No. 11/07 NERC/Centre for Ecology & Hydrology,, 2011.

Rothamsted-Research. Guide to the Classical and other Long-term Experiments, Datasets and Sample Archive. Rothamsted Research, Lawes Agricultural Trust Ltd, Harpenden UK, 2006. DOI: 10.23637/rothamsted-long-term-experiments-guide-2006

Semenov MA, Jamieson PD, Martre P. Deconvoluting nitrogen use efficiency in wheat: A simulation study. European Journal of Agronomy 2007; 26: 283-294.

Smith J, Smith P, Addiscott T. Quantitative methods to evaluate and compare Soil Organic Matter (SOM) Models. In: Powlson DS, Smith P, Smith JU, editors. Evaluation of Soil Organic Matter Models: Using Existing Long-Term Datasets. Springer Berlin Heidelberg, Berlin, Heidelberg, 1996, pp. 181-199.

Smith RI, Fowler D, Sutton MA, Flechard C, Coyle M. Regional estimation of pollutant gas dry deposition in the UK: model description, sensitivity analyses and outputs. Atmospheric Environment 2000; 34: 3757-3777.

Stamp LD. The land of Britain: the report of the Land Utilisation Survey of Britain. Survey., 1941.

Van Genuchten MT. A closed-form equation for predicting the hydraulic conductivity of unsaturated soils. Soil science society of America journal 1980; 44: 892-898.

Waines JG, Ehdaie B. Domestication and Crop Physiology: Roots of Green-Revolution Wheat. Annals of Botany 2007; 100: 991-998.

Table S6.1. The details of current treatments on the Broadbalk and Park Grass long term experiments used for model calibration and testing

| **Broadbalk*** | | **Park Grass**** | |
| --- | --- | --- | --- |
| Plot | Treatment since 1852 | Plot | Treatment since 1856 |
| 3 | nil | 3 | nil |
| 8 | N3PKNaMg | 13/2 | FYM+PM/FM |
| 15 | N5PKNaMg^†^ | 14/2 | N^¥^2PKNaMg (since 1858) |
| 16 (calibration) | N6PKNaMg^‡^ |  |  |
| 22 | FYM |  |  |

Nil: no fertilizer or manure applied

N2, N3, N4, N5, N6: 96, 144, 192, 240, 288 kg N ha^−1^ yr^-1^ as ammonium nitrate since1968; previously N as ammonium sulphate

N^¥^ 2.96 kg N ha^−1^ y^-1^ sodium nitrate.

†N2 up to 1967, N3 1968-1984 and N5 from 1985 onwards

^‡^ N4 1852-1864; un-manured 1865-1883, N*2 1884-1967, N2 1968 - 1984 and N6 from 1985 onwards.

P as triple super phosphate (35 kg P ha^−1^ yr^-1^)

KNaMg: non-limiting amounts

FYM:35 t ha^−1^ farmyard manure which contained a mean of 265 kg N ha^-1^. Applied each year to Broadbalk plot 22, and one year in four to Park Grass plot 13/2.

PM: Poultry manure applied one year in four to Park Grass plot 13/2 until 1999, supplying 65 kg N ha^-1^

FM: Fish meal applied one year in four to Park Grass plot 13/2 since 2003, supplying 65 kg N ha^-1^

*Continuous winter wheat 1843 until 1925, sequential fallowing 1926-1967. Other crops including potato, oats and fodder maize were introduced in rotation with wheat on part of the experiment since 1968. For more details, see <http://www.era.rothamsted.ac.uk/Broadbalk>. In this study, we used data from sections 3 and 5 (wheat in rotation) of the plots for model calibration and testing.

**Permanent grass since 1856. For more details, see [http://www.era.rothamsted.ac.uk/Park](mailto:shibu.muhammed@rothamsted.ac.uk)

Table S6.2. Statistical^*^ comparison of simulated and observed variables for Broadbalk (plot 16) under calibration.

| Treatments | $n$ | $r$ | $M$ | $RMSE$^±^ |
| --- | --- | --- | --- | --- |
| Wheat yield (Mg ha‾^1^) | 148 | 0.85 | −1.5 | 1.9 (63%) |
| Soil organic carbon (Mg C ha‾^1^) | 11 | 0.42 | 0.47 | 1.0 (3%) |
| Total soil nitrogen (Mg N ha‾^1^) | 12 | 0.03 | 0.39 | 0.4 (12%) |

Statistical analysis carried out using ModEval ([Smith et al. (1996](https://rezatecltd.sharepoint.com/sites/Shibufiles/Shared%20Documents/LTLS%20paper1/Submission/Supplementary%20doc%206.docx#_ENREF_21) and [Loague and Green (1991](https://rezatecltd.sharepoint.com/sites/Shibufiles/Shared%20Documents/LTLS%20paper1/Submission/Supplementary%20doc%206.docx#_ENREF_14)).

^*^Statistics: r, correlation coefficient; M, mean difference (observed-modelled); RMSE, root mean square error (best fit = 0).

^±^Values in parenthesis shows relative RMSE = RMSE/Observed.

Table S6.3. Statistical^*^ assessment of the model comparing simulated and observed variables for Broadbalk LTE, 1852 to 2010.

| Treatments | $n$ | $r$ | $M$ | $RMSE$^±^ |
| --- | --- | --- | --- | --- |
| *Wheat yield* | | | | |
| Plot 3 | 148 | 0.45 | 0.02 | 0.7 (52%) |
| Plot 8 | 148 | 0.76 | −1.4 | 1.9 (63%) |
| Plot 15 | 148 | 0.87 | −1.6 | 2.0 (70%) |
| Plot 22 | 148 | 0.74 | −1.5 | 2.1 (70%) |
| All plots combined | 592 | 0.80 | −1.0 | 1.7 (66%) |
| *Potato yield* | | | | |
| Plot 3 | 5 | 0.5 | 0.6 | 0.7 (36%) |
| Plot 8 | 5 | 0.8 | -0.9 | 1.4 (18%) |
| Plot 15 | 5 | -0.6 | -0.8 | 2.3 (31%) |
| Plot 22 | 5 | -0.1 | 0.2 | 5.6 (66%) |
| All plots combined | 20 | 0.8 | -0.2 | 1.9 (30%) |
| *Fodder maize* | | | | |
| Plot 3 | 4 | -0.98 | -1.4 | 1.9 (71%) |
| Plot 8 | 4 | -0.6 | 3.1 | 5.1 (42%) |
| Plot 15 | 4 | -0.7 | 1.1 | 3.6 (32%) |
| Plot 22 | 4 | -0.5 | 0.2 | 9.0 (69%) |
| All plots combined | 16 | 0.63 | 0.8 | 4.0 (38%) |
| *Soil organic carbon* | | | | |
| Plot 3 | 10 | -0.03 | −3.1 | 3.7 (16%) |
| Plot 8 | 10 | -0.49 | -0.04 | 3.3 (11%) |
| Plot 15 | 10 | 0.16 | 0.26 | 2.0 (7%) |
| Plot 22 | 12 | 0.93 | -1.8 | 4.5 (6%) |
| All plots combined | 53 | 0.99 | -1.7 | 3.6 (10%) |
| *Soil total nitrogen* | | | | |
| Plot 3 | 10 | 0.43 | 0.09 | 0.2 (9%) |
| Plot 8 | 10 | -0.42 | −0.39 | 0.5 (17%) |
| Plot 15 | 6 | 0.04 | 0.33 | 0.4 (12%) |
| Plot 22 | 12 | 0.88 | -0.36 | 0.7 (10%) |
| All plots combined | 50 | 0.97 | 0.11 | 0.5 (13%) |

Statistical analysis carried out using ModEval ([Smith et al. (1996](https://rezatecltd.sharepoint.com/sites/Shibufiles/Shared%20Documents/LTLS%20paper1/Submission/Supplementary%20doc%206.docx#_ENREF_21) and [Loague and Green (1991](https://rezatecltd.sharepoint.com/sites/Shibufiles/Shared%20Documents/LTLS%20paper1/Submission/Supplementary%20doc%206.docx#_ENREF_14)).

^*^Statistics: r, correlation coefficient; M, mean difference (observed-modelled); RMSE, root mean square error (best fit = 0).

^±^Values in parenthesis shows relative RMSE = RMSE/Observed.

Table S6.4. Statistical^*^ assessment of the model comparing simulated and observed variables for Park Grass LTE (1856 – 2009).

| Treatments | $n$ | $r$ | $M$ | $RMSE$^±^ |
| --- | --- | --- | --- | --- |
| *Park Grass* | | | | |
| *Grass yield (cut 1 + cut 2)* | | | | |
| Plot 3 | 154 | 0.28 | −0.9 | 1.3 (71%) |
| Plot 13/2 | 154 | 0.26 | −2.3 | 2.9 (55%) |
| Plot 14/2^†^ | 152^†^ | 0.47 | −1.7 | 2.1 (37%) |
| All plots combined | 460 | 0.80 | -0.7 | 1.3 (31%) |
| *Soil organic carbon* | | | | |
| Plot 3 | 3 | -1.0 | 25.6 | 30 (35%) |
| Plot 13/2 | 3 | 0.88 | −2.4 | 6.3 (7%) |
| Plot 14/2 | 3 | −1.0 | 5.4 | 12.2 (15%) |
| All plots combined | 9 | 0.05 | 10 | 19 (22%) |
| *Soil total nitrogen* | | | | |
| Plot 3 | 8 | 0.35 | 2.0 | 2.1 (27%) |
| Plot 13/2 | 3 | −0.7 | −0.9 | 1.0 (14%) |
| Plot 14/2 | 3 | -0.69 | 0.18 | 0.3 (4%) |
| All plots combined | 14 | -0.37 | 1.3 | 1.9 (26%) |

Statistical analysis carried out using ModEval ([Smith et al. (1996](https://rezatecltd.sharepoint.com/sites/Shibufiles/Shared%20Documents/LTLS%20paper1/Submission/Supplementary%20doc%206.docx#_ENREF_21) and [Loague and Green (1991](https://rezatecltd.sharepoint.com/sites/Shibufiles/Shared%20Documents/LTLS%20paper1/Submission/Supplementary%20doc%206.docx#_ENREF_14)).

^*^Statistics: r, correlation coefficient; M, mean difference (observed-modelled); RMSE, root mean square error (best fit = 0).

^±^Values in parenthesis shows relative RMSE = RMSE/Observed.

^†^Plot 14 started in 1858 and the others in 1856

**Grass**

**Land cover**

**Arable**


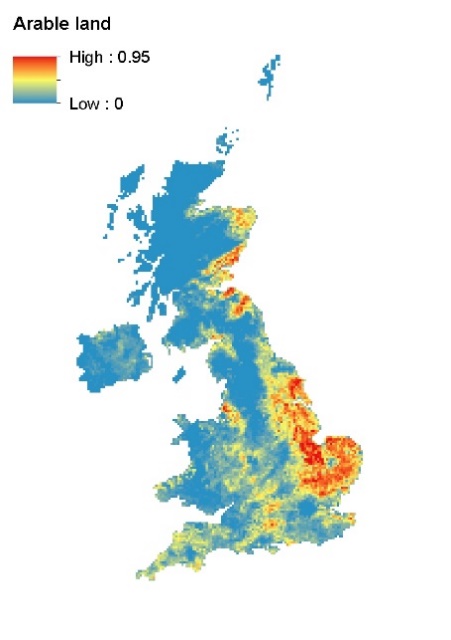

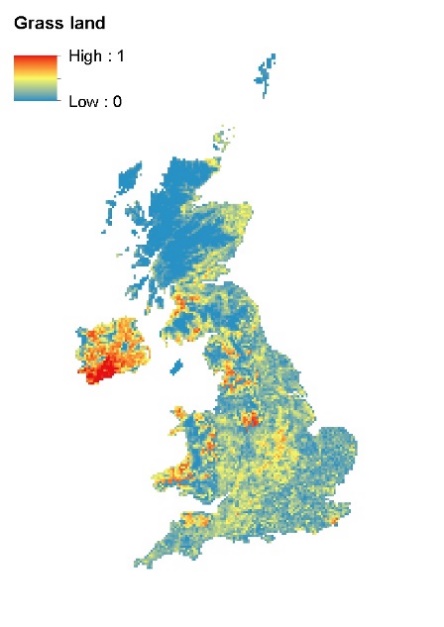


**Soil organic carbon**


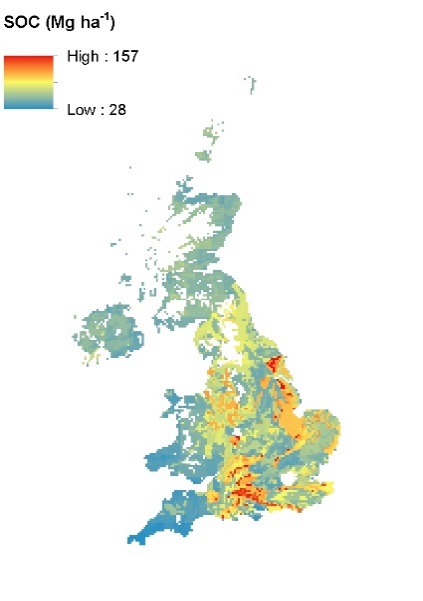

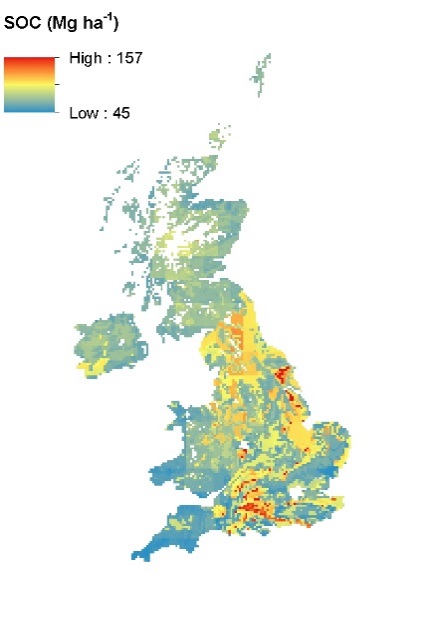


**Inorganic phosphorus**


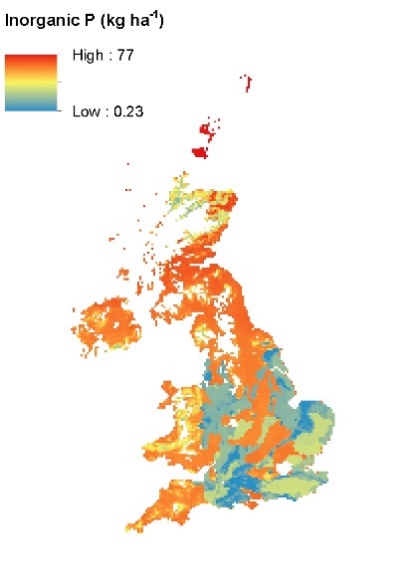

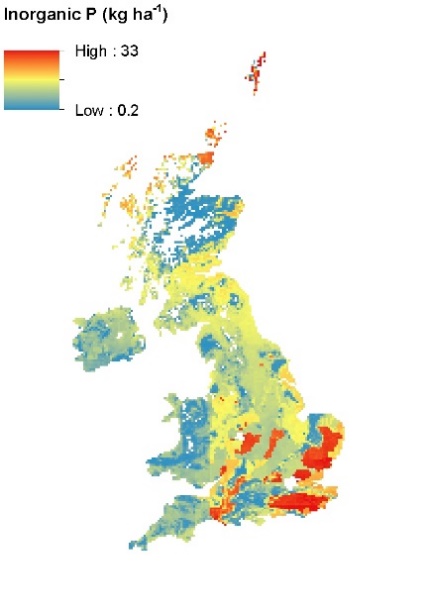


Figure S2.1. Landcover (as fraction of pixel area), initial soil organic carbon (Mg ha^−1^) and inorganic phosphorus (kg ha^−1^) initialised for arable and improved grassland from the outputs of semi-natural model (Davies et al., *in prep*) at the time of transition to agriculture in 1800.

Figure S2.2 Area under different landcovers during the time course of 1800-2007

**Old (before 1970)**

**Current (after 1970) 1970)**

**Grain**

**Straw yield**

**Harvest index**

**Aboveground biomass biomass**

Figure S6.1. Partitioning of biomass among different crop organs as a function of developmental stage (DVS) for the current ([Boons - Prins et al., 1993](https://rezatecltd.sharepoint.com/sites/Shibufiles/Shared%20Documents/LTLS%20paper1/Submission/Supplementary%20doc%206.docx#_ENREF_4)) and old varieties (before 1970) of wheat determined by calibration using Broadbalk data (plot 16) and the results of calibration for yield, straw, aboveground biomass and harvest index.

**Plot 22 (FYM35 t ha^−1^)**

**Plot 15 (96 and 240 kg N ha^−1^)**

**Plot 3 (nil)**

**Plot 8 (144 kg N ha-1)**

Figure S6.2. Comparison of simulated and observed wheat yields under different fertilizer N treatments in the Broadbalk LTE.

Figure S6.3. Simulated and observed soil organic carbon and soil total nitrogen under different fertilizer nitrogen treatments in the Broadbalk LTE.

Figure S6.4. Observed and simulated mean average annual loss of NO_3_-N during 1991-1998 under the Broadbalk LTE. Observed data from [Goulding et al. (2000](https://rezatecltd.sharepoint.com/sites/Shibufiles/Shared%20Documents/LTLS%20paper1/Submission/Supplementary%20doc%206.docx#_ENREF_13)).

**Plot 14/2 (96 kg N ha^-1^)**

**Plot 13/2 (FYM35 t ha^−1^)**

**Plot 3 (nil)**

Figure S6.5. Simulated and observed grass cut yields under different nitrogen treatments at the Park Grass. LTE.

Figure S6.6. Simulated and observed soil organic carbon and soil total nitrogen under different nitrogen treatments in Park Grass LTE.
