# Supplementary material for: Impact of two centuries of intensive agriculture on soil carbon, nitrogen and phosphorus cycling in the UK
Source: Sci Total Environ. 2018 Sep 1;634:1486–504. doi: 10.1016/j.scitotenv.2018.03.378 (PMC5981008; doi:10.1016/j.scitotenv.2018.03.378)
Supplement: Appendix I — Description of different processes in Roth-CNP model. [file mmc2.docx]

## **Appendix. I. Description of different processes in Roth-CNP model.**

| Processes | Model used | Description |
| --- | --- | --- |
| Soil organic C, N and P | Landscape model | Relevant equations can be found at section 2.3 of Coleman et al., 2017. To initialise SOC, N and P the model was not spun to equilibrium but initialised with the outputs from N14CP. The decomposition rate parameters were adapted to monthly timestep. Temporary pools of dissolved organic C, N and P will be active only when organic manures are added. |
| Nitrification | $N_{NO3}=k_{NO3} {ft}_{N} f{((\theta-\theta_{WP})/(\theta}_{FC}-\theta_{WP})) {fpH}_{N}$  Where $N_{NO3}$ is the rate of nitrification (kg ha d‾^1^), $k_{NO3}$ is the relative rate of nitrification (d‾^1^), ${ft}_{N}$, $f{((\theta-\theta_{WP})/(\theta}_{FC}-\theta_{WP}))$, ${fpH}_{N}$ are the rate modifiers for nitrification as a function of temperature, relative water content and pH.  $\theta,\theta_{FC}$, and $\theta_{WP}$ are soil volumetric water content at time t, at field capacity and at permanent wilting point. | As described by Godwin and Allan 1991; |
| Denitrification | $D_{NO3}=k_{dNO3} {ft}_{d} {f(\theta/\theta}_{sat)} {fpH}_{d}$  Where $D_{NO3}$ is the rate of denitrification (kg ha d‾^1^), $k_{dNO3}$ is the relative rate of denitrification (d‾^1^), ${ft}_{d}$, $f(\theta/\theta_{sat})$, ${fpH}_{N}$ are the rate modifiers for nitrification as a function of temperature, water filled pore space and pH.$\theta,\mathrm{and}\theta_{Sat}$ are soil volumetric water content at time *t* and water content at saturation, respectively. | The model is based on Godwin and Allan 1991 and rate modifiers were based on DelGrosso et al, 2000. |
| N_2_O emission | Not estimated | Not calculated separately as in Landscape model, but is considered as part of denitrification |
| Biological N fixation | ${Nfix}_{rate}={Nfix}_{max} f_{T} f_{m} f_{N}$  where${Nfix}_{rate}$ and ${Nfix}_{max}$ are the actual and maximum rates of BNF (g N m^−2^ month^−1^). *f_T,_ f_m_ and* *f_N_* are rate modifying factors for temperature, soil moisture and inorganic N respectively. | Liu et al., 2013 |
| P fixation | Landscape model | As described in section 2.5 of Coleman et al., 2017 |
| P release | Landscape model | As described in section 2.5 of Coleman et al., 2017 |
| N and P runoff | Landscape model | As described in section 2.4 of Coleman et al., 2017. However, surface (20 mm) water and nutrient concentration in the Roth-CNP is calculated as a function of soil depth. |
| NO_3_-N and P leaching | Landscape model | As described in section 2.4 of Coleman et al., 2017. Nutrient leaching depends on the nutrient content (kg ha‾^1^) and soil moisture (mm) of the profile and the drainage rate (mm month‾^1^). |
| Soil water | Not estimated | Soil moisture content (mm), runoff (mm month‾^1^), drainage (mm month‾^1^) and actual evapotranspiration (mm month‾^1^) are estimated by the hydrology model (Bell et al., *in prep*) |
| Biomass production | Landscape model | As described in section 2.7 of Coleman et al.,2017 |
| Crop development | Crop or grass specific fixed DVS | Estimated based on the daily simulation results of the Landscape model |
| Water stress | $W_{rf}=\frac{A_{ET}}{P_{ET}}$  Where $W_{rf}$ is the water stress, $A_{ET}$ and $P_{ET}$ are the actual and potential evapotranspiration (mm month‾^1^). | In Landscape model, water stress is calculated as the ratio of actual to potential transpiration, but Roth-CNP model does not separate the evapotranspiration into evaporation and transpiration. |
| Nutrient uptake | Landscape model, LINTUL3 | The uptake of plant nutrient (N and P) is determined by the crop demand and the supply of these nutrients by soil. In Roth-CNP, crop nutrient demand for various crops as a function of DVS is taken from the parameter set for LINTUL models (http://models.pps.wur.nl/glossary/l); Shibu et al., 2010. |
| Nutrient stress | Landscape model, LINTUL3 | As described in section 2.7 of Coleman et al., 2017; Shibu et al., 2010. |

Coleman K, Muhammed SE, Milne AE, Todman LC, Dailey AG, Glendining MJ, et al. The Landscape Model: a model for exploring trade-offs between agricultural production and the environment. Science of the Total Environment 2017; 609: 1483-1499.

Godwin, D.C., Allan, Jones C., 1991. Nitrogen dynamics in soil-plant systems. Modeling Pant and Soil Systems. ASA-CSSA-SSSA, Madison.

Liu Y, Wu L, Watson CA, Baddeley JA, Pan X, Zhang L. Modeling Biological Dinitrogen Fixation of Field Pea with a Process-Based Simulation Model. Agronomy Journal 2013; 105: 670-678.

Shibu, M.E., Leffelaar, P.A., van Keulen, H., Aggarwal, P.K., 2010. LINTUL3, a simulation model for nitrogen-limited situations: Application to rice. Eur. J. Agron. 32, 255–271.
